# CO_2_ controls the oriented growth of metal-organic framework with highly accessible active sites

**DOI:** 10.1038/s41467-020-15200-4

**Published:** 2020-03-18

**Authors:** Fanyu Zhang, Jianling Zhang, Bingxing Zhang, Lirong Zheng, Xiuyan Cheng, Qiang Wan, Buxing Han, Jing Zhang

**Affiliations:** 1 Beijing National Laboratory for Molecular Sciences, CAS Key Laboratory of Colloid, Interface and Chemical Thermodynamics, CAS Research/Education Center for Excellence in Molecular Sciences, Institute of Chemistry, Chinese Academy of Sciences, 100190 Beijing, People’s Republic of China; 2 School of Chemical Science, University of Chinese Academy of Sciences, 100049 Beijing, People’s Republic of China; 3 Physical Science Laboratory, Huairou National Comprehensive Science Center, 101400 Beijing, People’s Republic of China; 4 Beijing Synchrotron Radiation Facility (BSRF), Institute of High Energy Physics, Chinese Academy of Sciences, 100049 Beijing, People’s Republic of China

**Keywords:** Green chemistry, Metal-organic frameworks

## Abstract

The production of 2D metal-organic frameworks (MOFs) with highly exposed active surfaces is of great importance for catalysis. Here we demonstrate the formation of MOF nanosheets by utilizing CO_2_ as a capping agent to control the oriented growth of MOF. This strategy has many advantages over the conventional methods. For example, it is template-free and proceeds at mild temperature (35 °C), CO_2_ can be easily removed by depressurization, and the properties of the MOF nanosheets can be well adjusted by changing CO_2_ pressure. Such a simple, rapid, efficient and adjustable route produces MOF nanosheets with ultrathin thickness (∼10 nm), small lateral size (∼100 nm) and abundant unsaturated coordination metal sites on surfaces. Owing to these unique features, the as-synthesized MOF nanosheets exhibit superior activity for catalyzing the oxidation reactions of alcohols.

## Abstract (web summary)

Producing two-dimensional metal-organic frameworks (MOFs) with highly exposed active surfaces is important for catalysis. Here, authors prepare MOF nanosheets by using CO_2_ as a capping agent to control the oriented growth of MOFs.

## Introduction

Metal-organic frameworks (MOFs) are a new type of crystalline porous materials that are self-assembled by the coordination of metal cations/clusters with organic linkers^1^, which have broad applications in gas storage and separation^2–4^, catalysis^5^, sensor^6^, drug delivery^7^, etc. Particularly, MOFs have exhibited promising catalytic potentials towards many kinds of reactions owing to their designable metal-oxo clusters bridging organic linkers, modifiable structure, and intrinsic porosities^8^. However, the catalytic activities of MOFs are still largely restricted for the low-mass permeability, poor conductivity, and blockage of active metal centers by organic ligands^9^. Ultrathinning three-dimensional (3D) MOFs into two-dimensional (2D) MOFs is an effective strategy to acquire high-performance catalysts because of the highly exposed active surfaces, superior electron transfer and facilitated mass transport of 2D MOFs^9–15^. Up to now, the methods for synthesizing 2D MOFs can be classified into two groups: top-down and bottom-up strategies. The top-down exfoliation approach relies on the disintegration of 3D layered MOF solid^11,16–19^, usually suffering from high-energy consumption, fragmentation and morphological damage and low yields (typically < 15%)^9,20^. For the bottom-up strategy, 2D MOFs are synthesized directly from starting materials by solvothermal route using additives (e.g., surfactant, capping molecules, template) to control the oriented MOF growth^21–25^. The temperature is high (>80 °C) and the post-processing procedure is complex to remove the solid or liquid additives. It is of great importance to develop facile and efficient method for the synthesis of 2D MOFs, promisingly with highly accessible active sites that are desirable for catalysis.

Herein we propose a CO_2_-directed route for synthesizing MOF nanosheets. By utilizing CO_2_ as a capping agent for controlling the oriented growth of MOF, the MOF nanosheets with ultrathin thickness, small lateral size and highly accessible active sites were obtained. In comparison with the conventional methods for 2D MOFs synthesis, this strategy has many advantages. First, CO_2_ can direct the oriented growth of MOF by adsorbing on the specific facets of MOF crystal, involving no additional template. Second, CO_2_ accelerates MOF formation at mild temperature (35 °C). Third, the properties of the as-synthesized 2D MOFs can be well adjusted by changing CO_2_ pressure. Fourth, CO_2_ can be easily removed by depressurization. Moreover, the CO_2_ desorption from MOF creates abundant unsaturated sites on surfaces that are desirable for catalysis. The as-synthesized MOF nanosheets have shown highly efficient and reusable catalysts for oxidation reactions of a series of alcohols.

## Results

### Synthesis and structural characterizations of Cu(BDC) nanosheets

**Fig. 1:**
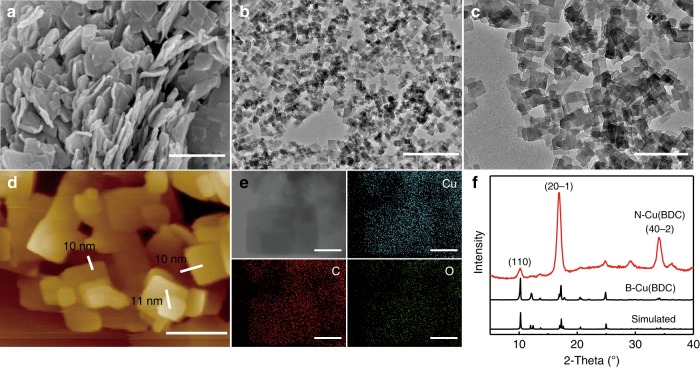
Structural characterizations of N-Cu(BDC). **a**–**e** SEM, TEM, AFM images, and EDS mappings of N-Cu(BDC) synthesized in CO_2_/methanol solution at 7.38 MPa and 35 °C with 0.1 mL of TEA for 24 h; **f** XRD patterns of N-Cu(BDC), B-Cu(BDC), and simulated XRD pattern of bulk Cu(BDC). Scale bars: 500 nm in **a** and **c**, 1 μm in **b**, 200 nm in **d** and 50 nm in **e**.

For the synthesis of Cu(BDC) (BDC = 1,4-benzenedicarboxylate) nanosheets, CO_2_ was charged into the methanol solution of Cu(NO_3_)_2_·3H_2_O, terephthalic acid and triethylamine (TEA) at 35 °C until a desired pressure was reached (see device in Supplementary Fig. 1). After reaction for a certain time, the solid product was obtained by depressurization and washing. Figure 1 shows the characterizations for the product obtained at 7.38 MPa and reaction for 24 h. Scanning electron microscopy (SEM) and transmission electron microscopy (TEM) images show the formation of square nanosheets with edge length of ∼120 nm (Fig. 1a–c). The thickness of nanosheets is ∼10 nm, as characterized by atomic force microscope (AFM, Fig. 1d and Supplementary Fig. 2). Elemental mapping and energy-dispersive X-ray spectroscopy (EDS) images reveal that Cu, C and O elements distribute evenly in the product (Fig. 1e and Supplementary Fig. 3). X-ray diffraction (XRD) pattern mainly exhibits (20-1) and (40-2) crystallographic planes of Cu(BDC) (Fig. 1f), suggesting that these planes are all perpendicular to the stacking direction of the layers. High-resolution TEM image gives further support that the exposed surface of nanosheet is (20-1) facet (Supplementary Fig. 4). In other words, the flat top and bottom surfaces of Cu(BDC) are bounded by (20-1) facets^26^. For comparison, the Cu(BDC) was synthesized by the conventional solvothermal route, which shows cubic crystals with edge dimensions ranging from 0.5 to 10 µm (Supplementary Fig. 5). Its XRD pattern and relative intensities of the diffractions match well with the simulated diffractions of bulk Cu(BDC) (Fig. 1f)^27^. The Cu(BDC) nanosheets synthesized in CO_2_ and the bulk Cu(BDC) produced by solvothermal route are denoted as N-Cu(BDC) and B-Cu(BDC), respectively.

### Fine structure of N-Cu(BDC)

**Fig. 2:**
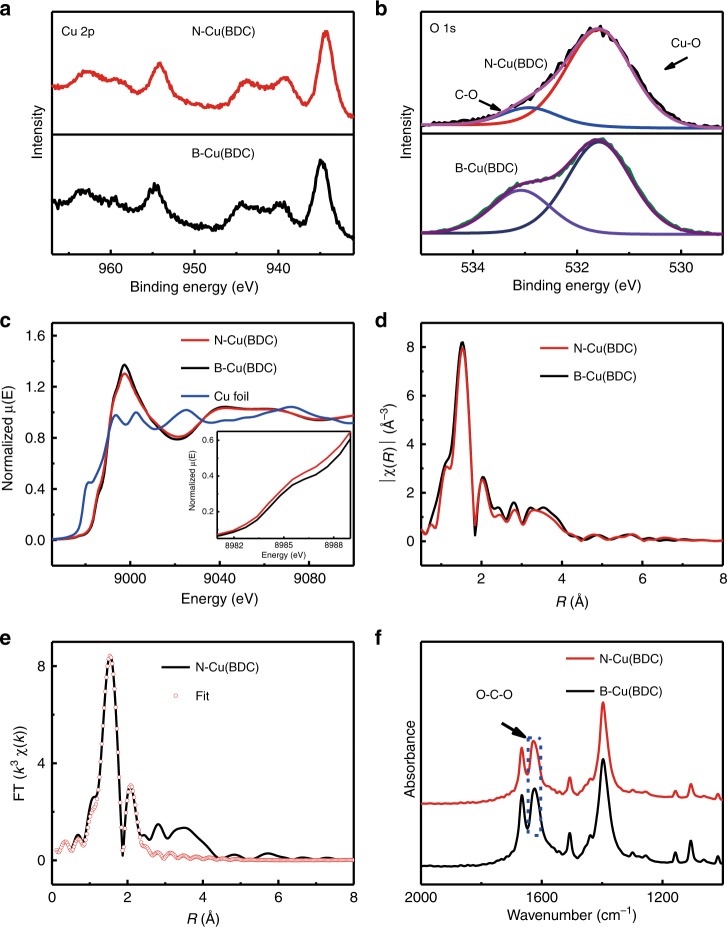
Fine structure of N-Cu(BDC). **a**, **b** Cu 2p and O 1s XPS spectra of N-Cu(BDC) synthesized in CO_2_/methanol at 7.38 MPa and 35 °C with 0.1 mL of TEA for 24 h and B-Cu(BDC); **c** XANES spectra at Cu K-edge of B-Cu(BDC), N-Cu(BDC) and Cu foil (inset is the expanded pre-edge region); **d** EXAFS spectra of B-Cu(BDC) and N-Cu(BDC); **e** EXAFS fitting curve of N-Cu(BDC); **f** FT-IR spectra of B-Cu(BDC) and N-Cu(BDC).

The insightful chemical and structural information of N-Cu(BDC) were investigated by X-ray photoelectron spectroscopy (XPS) (Fig. 2a, b and Supplementary Fig. 6). From the high resolution of Cu 2p spectra (Fig. 2a), the main peak of Cu (934.5 eV) of N-Cu(BDC) shifts to lower binding energy (0.4 eV) as compared with that of B-Cu(BDC). It indicates the presence of low valence state of Cu in N-Cu(BDC)^28–30^, which can be ascribed to the oxygen vacancies or linker missing in framework structure along with the formation of N-Cu(BDC). The O 1s spectrum of N-Cu(BDC) presents two curve-fitted peaks corresponding to two different types of oxygen species (Fig. 2b). The band at 531.9 eV corresponds to Cu-O species, of which the relative strength is obviously higher than that of B-Cu(BDC). It suggests that more Cu-O clusters are exposed on the surface of Cu(BDC) nanosheet. To further detect the local structure of N-Cu(BDC) at atomic level, the synchrotron X-ray absorption fine structure (XAFS) was determined. For Cu K-edge X-ray absorption near-edge structure (XANES) (Fig. 2c), the N-Cu(BDC) exhibits similar spectrum features to those of B-Cu(BDC), suggesting that their main structures are the same. The absorption edge of N-Cu(BDC) is at 8989.3 eV, which is between those of B-Cu(BDC) (8989.9 eV) and Cu foil (8979.0 eV). It indicates the existence of Cu^δ+^ (0 < *δ* < 2) species in N-Cu(BDC). From extended X-ray absorption fine structure (EXAFS) spectra (Fig. 2d), the peak centered at 1.53 Å for the N-Cu(BDC) shows an intensity decrease, which further suggests the formed Cu(BDC) nanosheets with lower coordination number^30^. By fitting for EXAFS (Fig. 2e, Supplementary Fig. 7, and Table 1), it is confirmed that the coordination number of Cu is 4.0, which is less than that of B-Cu(BDC) (five oxygen atoms around one Cu atom)^27^. Fourier-transform infrared spectroscopy (FT-IR) proves that carboxylate groups of BDC^2−^ are coordinated to Cu (II) ions in N-Cu(BDC) (Supplementary Fig. 8). Moreover, the absorption of -COO^−^ for N-Cu(BDC) centers at 1628 cm^−1^ (Fig. 2f), which is higher than that in B-Cu(BDC) (1624 cm^−1^). It can be ascribed to the more freedom of oxygen atoms in N-Cu(BDC).

### Evolution of N-Cu(BDC) with reaction time

**Fig. 3:**
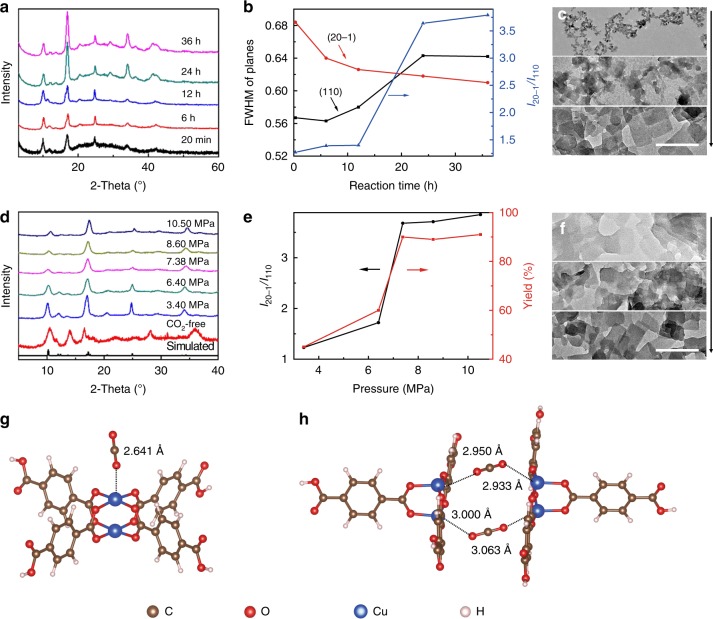
Tunability and formation mechanism of N-Cu(BDC). **a** XRD patterns of the Cu(BDC) synthesized in CO_2_/methanol solution at 7.38 MPa and 35 °C with 0.1 mL of TEA at different time; **b** Dependence of FWHM of planes and the relative intensity of (20-1) plane to (110) plane (*I*_20-*1*_ */I*_110_) on reaction time; **c** TEM images of the Cu(BDC) synthesized with time of 20 min, 12 and 36 h; **d** XRD patterns of the Cu(BDC) synthesized in CO_2_/methanol solution at 35 °C and 24 h with 0.1 mL of TEA at different pressures; **e** Dependence of the relative intensity of (20-1) plane to (110) plane (*I*_20-1_/*I*_110_) and the yield of Cu(BDC) on pressure; **f** TEM images of Cu(BDC) synthesized at 3.40, 6.40 and 8.60 MPa, respectively; **g** Structural units of Cu(BDC) and CO_2_ along [20-1] axis; **h** Structural units of Cu(BDC) and CO_2_ along (20-1) plane. Scale bar: 300 nm in **c** and **f**.

The formation of N-Cu(BDC) in CO_2_ is very interesting and the underlying mechanism was investigated by different experiments. First, the evolution of the N-Cu(BDC) was investigated by varying reaction time, the other conditions being the same with those above. As the reaction time is as short as 20 min, the XRD peak positions and relative intensities of the product agree well with those of the reported Cu(BDC), but the crystallinity is poor (Fig. 3a). With prolonged reaction time, the crystallinity is enhanced and 24 h is enough to get the well crystallized product. The full-width half-maximum (FWHM) values of (110) peaks of the samples synthesized at different time increase with time, while those of (20-1) peaks decline with reaction time (Fig. 3b). The relative intensities of (20-1) plane to (110) plane enhance with time (Fig. 3b). These results indicate that the (20-1) plane grows faster than (110) plane, thus resulting in the formation of Cu(BDC) nanosheets at prolonged reaction time. The sample synthesized at 20 min presents small nanoparticles in average size of 10 nm (Fig. 3c and Supplementary Fig. 9). With prolonged reaction time, it changes to the irregular nanosheets with size of ∼50 nm at 12 h (Fig. 3c) and regular nanosheets (∼120 nm) at 24 and 36 h (Figs. 1c and 3c). The yields of the materials increase with reaction time and can reach 90% when the reaction time extends to 24 h (Supplementary Fig. 10).

### Effect of CO_2_ pressure on N-Cu(BDC) formation

The effect of CO_2_ pressure on the N-Cu(BDC) formation was investigated by varying pressure. The reaction time was fixed at 24 h. In the absence of CO_2_ (at atmospheric pressure), Cu(BDC) could not be formed in pure methanol, suggesting that CO_2_ plays a key role on the Cu(BDC) nanosheets formation (Fig. 3d and Supplementary Fig. 11). The main peaks in XRD patterns of the products obtained at pressure of 3.40, 6.40, 8.60 and 10.50 MPa match well with those of the simulated XRD of Cu(BDC) (Fig. 3d), indicating the formation of Cu(BDC) in presence of CO_2_. Obviously, the relative intensity of (20-1) to (110) is dependent of pressure. It increases slowly at initial stage and increases sharply in pressure range of 6.40–7.38 MPa (Fig. 3e). It means that more CO_2_ is favorable for accelerating the growth of (20-1) plane and simultaneously restrains the (110) plane of Cu(BDC). At the same time, the dependence of the Cu(BDC) yield with pressure shows a similar trend to that of the relative intensity of (20-1) plane to (110) plane (Fig. 3e). From TEM (Fig. 3f and Supplementary Fig. 12) and SEM images (Supplementary Figs. 13 and 14), it is clear that high-pressure favors the formation of nanosheets. The average sizes of the nanosheets obtained at 7.38, 8.60, and 10.50 MPa are 120, 102, and 96 nm, with thicknesses of 10, 10, and 7 nm, respectively (Supplementary Figs. 15–19). Evidently, the size of the N-Cu(BDC) decreases with increasing CO_2_ pressure. It is known that CO_2_ is well solubilized in organic solvent to cause large volume expansion and lowered viscosity, especially at high pressure (Supplementary Fig. 20). Therefore, the CO_2_-expanded solution at higher pressure is favorable for producing N-Cu(BDC) with smaller size^31–34^.

### Effect of temperature on Cu(BDC) formation

To detect the effect of temperature on N-Cu(BDC) formation, the synthesis was also carried out at 25 °C and 45 °C, respectively, while the pressure was fixed at 7.38 MPa. XRD, SEM, and TEM results reveal the formation of Cu(BDC) nanosheets (Supplementary Figs. 21–24), which have similar size with those synthesized at 35 °C. It means that the size of Cu(BDC) nanosheets is independent of temperature. The yields of Cu(BDC) nanosheets synthesized at 25, 35, and 45 °C are 69%, 90%, and 95%, respectively. Obviously, higher temperature accelerates the production of Cu(BDC) nanosheets.

### Effects of other factors on Cu(BDC) formation

To get more information on the formation mechanism for Cu(BDC) nanosheets, a series of experiments were carried out. First, Ar or N_2_ was used as an alternative of CO_2_ to synthesize Cu(BDC) in methanol. The gas pressure and reaction time were fixed at 7.30 MPa and 24 h, respectively, the other experimental conditions being the same with above. The results show that Cu(BDC) nanosheets could not be formed by using Ar or N_2_ (Supplementary Figs. 25 and 26). Second, the Cu(BDC) synthesis was carried out in CO_2_/acetonitrile and CO_2_/ethanol solutions at 7.38 MPa and 35 °C, respectively. The Cu(BDC) nanosheets that are similar to those formed in CO_2_/methanol solution were obtained (Supplementary Figs. 27 and 28). It proves that it is indeed CO_2_ that dominates the formation of Cu(BDC) nanosheets. Third, to detect whether the carbonation of CO_2_ in water has an effect on N-Cu(BDC) formation, the MOF synthesis was carried out in Na_2_CO_3_/methanol and NaHCO_3_/methanol solutions (without CO_2_), respectively. The obtained products present large irregular agglomerates (Supplementary Figs. 29 and 30). Therefore, the effect of CO_2_ ionization in water on the N-Cu(BDC) formation can be ruled out. Fourth, the effect of TEA dosage on the formation of N-Cu(BDC) in CO_2_/methanol solution was studied. The pressure and temperature were fixed at 7.38 MPa and 35 °C, respectively. It was found that the enough amount of TEA, which plays a role for H_2_BDC deprotonation, is needed for accelerating the reaction between Cu^2+^ and the deprotonated BDC^2−^ to form framework building blocks and the further nanosheet formation (Supplementary Figs. 31 and 32).

### Density functional theory (DFT) calculations

To further understand the underlying mechanism for the CO_2_-produced Cu(BDC) nanosheets, DFT calculations were performed. The interaction distance between O in CO_2_ and Cu ion along [20-1] axis was calculated to be 2.641 Å, while that along (20-1) plane is 2.950 Å belonging to non-bonded interaction (Fig. 3g, h). It indicates that CO_2_ is more easily adsorbed on the (20-1) crystal facet, so as to forbid the stacking of (20-1) crystal facets along [20-1] axis. (20-1) plane is the dominantly exposed crystal facets and CO_2_ has no effect on the coordination between organic ligands and Cu^2+^ along (20-1) plane (Supplementary Fig. 33). It is worth noting that the interactions of Cu(BDC) with methanol along (20-1) plane and [20-1] axis are almost equivalent (Supplementary Fig. 34). Therefore, it can be understood that pure methanol has no effect to direct the 2D growth of Cu(BDC) and cannot produce nanosheets (Supplementary Fig. 11).

### Formation mechanism of N-Cu(BDC)

Based on the above results, a possible mechanism for the formation of Cu(BDC) nanosheets by CO_2_ is proposed (Supplementary Fig. 35). First, Cu^2+^ react with the deprotonated BDC^2−^ by TEA to structure the nanosized framework building blocks. Subsequently, CO_2_ molecule interacts with Cu^2+^ on (20-1) plane, which forbids the stacking of (20-1) crystal facets along [20-1] axis. The viscosity-lowering effect caused by CO_2_ would promote mass transfer and accelerate the formation of MOF nanocrystals. After reaction, CO_2_ is released by depressurization, during which the desorption of CO_2_ leaves abundant uncoordinated sites on the surface of Cu(BDC) nanosheets. The as-synthesized N-Cu(BDC) has a much improved CO_2_ adsorption ability than the bulk Cu(BDC) produced in pure methanol (Supplementary Fig. 36). The above result that high CO_2_ pressure benefits to the formation of Cu(BDC) nanosheets can be well understood (Fig. 3d–f). At low pressure, the amount of CO_2_ solubilized in reaction system is small, as well as the CO_2_ adsorbed on (20-1) crystal facets. Consequently, CO_2_ cannot control the oriented growth of Cu(BDC) effectively. The massive dissolution of CO_2_ in reaction system makes the sufficient adsorption of CO_2_ on (20-1) crystal facets when the pressure reaches 7.38 MPa, so as to direct the 2D growth of Cu(BDC) crystal. At the same time, the viscosity-lowering effect caused by CO_2_ is more obvious at higher pressure, which favors the production of Cu(BDC) nanosheets with larger yield.

### Catalytic activities of N-Cu(BDC)

**Fig. 4:**
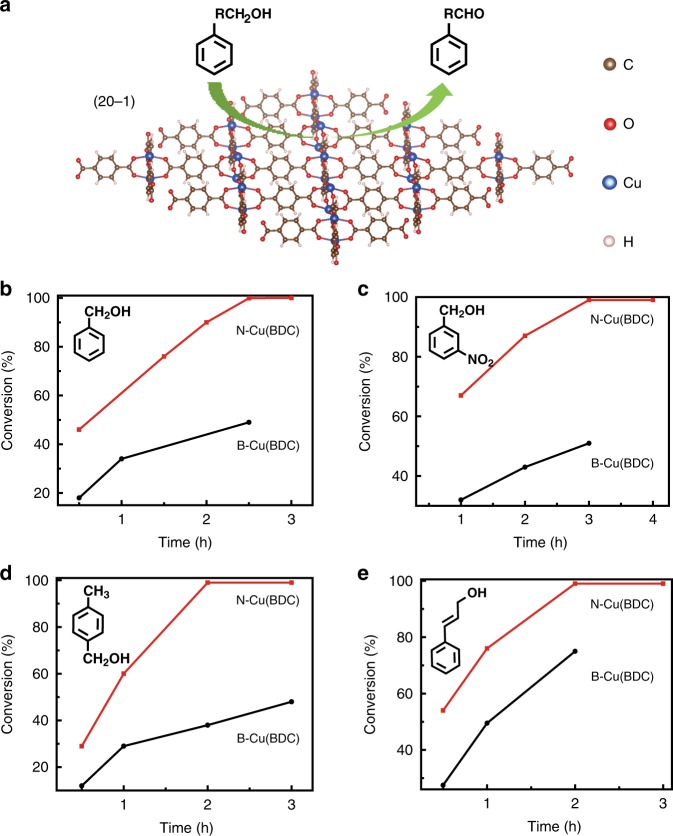
Catalytic activities of N-Cu(BDC) and B-Cu(BDC). **a** Aerobic oxidation of benzyl alcohol on N-Cu(BDC) synthesized in CO_2_/methanol solution at 7.38 MPa and 35 °C with 0.1 mL of TEA for 24 h; **b**–**e** Time conversion plot for the aerobic oxidation of benzyl alcohol, 3-nitrobenzyl alcohol, 4-methylbenzyl alcohol, and cinnamyl alcohol to corresponding products catalyzed by N-Cu(BDC) and B-Cu(BDC), respectively.

The N-Cu(BDC) was used to catalyze the oxidation of benzyl alcohol to benzaldehyde (Fig. 4a), which is an important reaction both in organic synthesis and chemical industry^35–37^. The catalytic reaction was performed under 75 °C, using 2,2,6,6-teramethyl-piperidine-1-oxyl (TEMPO) as a co-catalyst, Na_2_CO_3_ as base, oxygen as oxidant and N,N-dimethylformamide (DMF) as solvent. First of all, a control experiment for benzyl alcohol oxidation in the presence of only TEMPO (without N-Cu(BDC)) was carried out, which shows that benzyl alcohol cannot be converted in 2.5 h. With the addition of N-Cu(BDC), the conversion of benzyl alcohol to benzaldehyde reaches 99% at 2.5 h, with selectivity of >99% (Fig. 4b). In sharp contrast, the yield of benzaldehyde is only 49% after a reaction time of 2.5 h as catalyzed by B-Cu(BDC) at the same conditions. The catalytic activity of N-Cu(BDC) for the oxidation of benzyl alcohol to benzaldehyde is also higher than the mesoporous Cu_3_(BTC)_2_ under similar reaction conditions^34^ (Supplementary Table 2). After reaction, the N-Cu(BDC) was recovered and its reusability for the reaction was tested using benzyl alcohol as a model substrate. The conversion of benzyl alcohol remains unchanged at the 5th run for catalysis (Supplementary Fig. 37). No obvious difference was observed for the XRD pattern and TEM images of fresh Cu(BDC) and that after reused for five runs (Supplementary Fig. 38), indicating the well preserved structural integrity of N-Cu(BDC) catalyst. Inductively coupled plasma atomic emission spectrometer (ICP-AES) was used to determine whether there was Cu leached to DMF after reaction for five runs. No copper ions were detected, confirming the stability of N-Cu(BDC) for the oxidation reaction in DMF.

The catalytic activities of the as-synthesized N-Cu(BDC) were tested for the aerobic oxidation of various alcohols (Fig. 4c–e). 3-nitrobenzyl alcohol, 4-methylbenzyl alcohol and cinnamyl alcohol converted completely to the corresponding aldehydes in 3, 2, and 2 h, respectively. In contrast, the aldehyde yields catalyzed by B-Cu(BDC) only reach 51%, 38%, and 75% for these three alcohols at reaction time of 3, 2, and 2 h, respectively. The aerobic oxidations of furfuryl alcohol, 2-naphthalenemethanol, 1-phenylethanol, and 1-(4-methoxyphenyl)ethanol as catalyzed by N-Cu(BDC) were also studied. At 2.5 h, these alcohols can be converted to their corresponding aldehydes with yields of 70%, 89%, 44%, and 40%, respectively (Supplementary Table 3), which are all higher than those catalyzed by the reported Cu_3_(BTC)_2_ at similar conditions^38^.

Moreover, the catalytic activities of Cu(BDC) catalysts synthesized in CO_2_/methanol solutions at different CO_2_ pressures and 35 °C for catalyzing the oxidation of benzyl alcohol to benzaldehyde were studied. The reaction conditions were the same with those above. As catalyzed by Cu(BDC) synthesized at 3.40 and 6.40 MPa, the yields of benzaldehyde are 62% and 85%, respectively, while the yields of benzaldehyde catalyzed by Cu(BDC) synthesized at pressures higher than 7.38 MPa is 99% (Supplementary Table 2). It is obvious that the Cu(BDC) synthesized at higher pressure is more active, which can be attributed to its smaller size and more exposed active sites for catalysis^39^.

## Discussion

The high catalytic activity of the N-Cu(BDC) synthesized in this work can be attributed to their smaller particle size and more unsaturated metal sites than that synthesized by hydrothermal route. First, the N-Cu(BDC) synthesized by CO_2_ has nanometer sizes in all the three dimensions, which is favorable to increasing the density of catalytic active sites. Second, the N-Cu(BDC) synthesized by CO_2_ has abundant unsaturated coordination Cu sites on surfaces that are available for catalysis^40–42^, which are superior to the dormant and fully coordinated framework metal ions of MOFs that are unavailable for catalysis. Owing to these unique features, the as-synthesized N-Cu(BDC) exhibits greatly enhanced activity for catalyzing the oxidation reactions of alcohols as compared with B-Cu(BDC).

The versatility of the CO_2_-directed route for the formation of other MOF nanosheets was investigated. The nanosheets of MOFs with different metal ions and organic ligands, i.e., Co(BDC), Cu(1,4-NDC) (1,4-NDC = 1,4-naphtalenedicarboxylate), bi-metal (Co, Ni)(BDC) were successfully synthesized (Supplementary Figs. 39–51). Moreover, the CO_2_-directed route can be applied to the large-scale synthesis of Cu(BDC) nanosheets (Supplementary Figs. 52 and 53).

In summary, we propose a CO_2_-directed route for controlling the oriented growth of MOF. This strategy produces MOF nanosheets with ultrathin thickness (∼10 nm), ultra-small lateral size (∼100 nm) and abundant unsaturated coordination metal sites on the surface of nanosheets. These combined features confer the as-synthesized MOF nanosheets many advantages for catalyzing chemical reactions, particularly facilitating the approach of reactant molecules to catalytically active sites. The proposed CO_2_ method is facile, rapid, adjustable, high-yielding, low-energy and environmentally benign for MOF material synthesis. We anticipate that it could be applied to the fabrication of other MOFs and MOF-based materials with desirable structures and functions.

## Methods

### Materials

Terephthalic acid (H_2_BDC, 99% purity), methyl sulfoxide-d6 (99.8% atom% D purity), cinnamyl alcohol (98% purity), 3-nitrobenzyl alcohol (>99% purity), 4-methylbenzyl alcohol (98% purity), and TEMPO (98% purity) were supplied by Beijing InnoChem Science & Technology Co., Ltd. CO_2_ ( >99.95% purity), water, N_2_ ( >99.95% purity), Ar (>99.999% purity), and O_2_ (99.99% purity) were purchased from Beijing Analysis Instrument Factory. TEA (A. R. grade), ethanol (A. R. grade), methanol (A. R. grade), DMF (A. R. grade), acetonitrile (A. R. grade), Na_2_CO_3_ (A. R. grade), and NaHCO_3_ (A. R. grade) were supplied by Beijing Chemical Works. CoCl_2_·6H_2_O (A. R. grade), Cu(NO_3_)_2_·3H_2_O (A. R. grade), and NiCl_2_·6H_2_O (A. R. grade) were brought from China National Medicines Co., Ltd. 1,4-Naphthalenedicarboxylic acid (H_2_NDC, 99% purity), 2-naphthalenemethanol (98% purity), 1-phenylethanol (97% purity), 1-(4-methoxyphenyl)ethanol (95% purity), and furfuryl alcohol (98% purity) were purchased from J&K Scientific Co., Ltd. Benzyl alcohol was supplied by Alfa Aesar China Co., Ltd. All these materials were used directly without further purification.

### Cu(BDC) synthesis

For a typical experiment, Cu(NO_3_)_2_·3H_2_O (0.0180 g), H_2_BDC (0.0170 g), TEA (0.1 mL) and methanol (5 mL) were added into autoclave. Then CO_2_ was charged into the autoclave under stirring to the desired pressure for 24 h at 35 °C. The precipitate was collected, washed by DMF and ethanol twice and dried at 80 °C in vacuum for 8 h. A series of control experiments were carried out. First, N_2_ or Ar was used as an alternative of CO_2_ for Cu(BDC) synthesis, while the other conditions were the same with those above. Second, Cu(BDC) was synthesized in CO_2_/ethanol and CO_2_/acetonitrile solutions, respectively, the other conditions being the same with those above for the synthesis in CO_2_/methanol solution (35 °C and 7.38 MPa). Third, the Cu(BDC) synthesis was conducted in NaHCO_3_/methanol and Na_2_CO_3_/methanol solution (without CO_2_), respectively. The amount of NaHCO_3_ or Na_2_CO_3_ was 20 mg, while the dosages of other reagents were the same with those above for the synthesis in CO_2_/methanol solution.

### Bulk Cu(BDC) synthesis

Bulk Cu(BDC) was synthesized following the hydrothermal synthesis described by Carson^27^. 0.5000 g of Cu(NO_3_)_2_·3H_2_O, 0.3500 g of H_2_BDC and 43 mL of DMF were mixed in a 100 mL round-bottom flask and refluxed at 100 °C for 24 h. The resulting powder was collected by centrifugation at 10,000 rpm and the solid was consecutively washed with DMF (20 mL each step) and ethanol (20 mL each step) for three times, respectively. The product was obtained after drying at 80 °C in vacuum for 8 h.

### Co(BDC) nanosheets synthesis

CoCl_2_·6H_2_O (0.0108 g) and H_2_BDC (0.0100 g) were dissolved into 4 mL DMF mixed with 0.25 mL water and 0.25 mL ethanol. The above solution was stirred for 30 min, followed by the addition of 0.1 mL TEA. Then CO_2_ was charged into the autoclave under stirring. The mixture was stirred at 7.38 MPa and 35 °C for 24 h. The precipitate was washed by DMF and ethanol twice and dried at 80 °C in vacuum for 8 h.

### Cu(1,4-NDC) nanosheets synthesis

Cu(NO_3_)_2_·3H_2_O (0.0180 g), H_2_NDC (0.0210 g), methanol (5 mL) and TEA (0.1 mL) were added into autoclave. CO_2_ was charged into the autoclave under stirring. Then the mixture was stirred at 7.38 MPa and 35 °C for 24 h. The precipitate was collected, washed by DMF and ethanol twice and dried at 80 °C in vacuum for 8 h.

### (Co, Ni)(BDC) nanosheets synthesis

CoCl_2_·6H_2_O (0.0108 g), NiCl_2_·6H_2_O (0.0108 g) and H_2_BDC (0.0180 g) were dissolved into 4 mL DMF mixed with 0.25 mL water and 0.25 mL ethanol. The above solution was stirred for 30 min, followed by the addition of 0.1 mL TEA. Then CO_2_ was charged into the autoclave under stirring. The mixture was stirred at 7.38 MPa and 35 °C for 24 h. The precipitate was washed by DMF and ethanol twice and dried at 80 °C in vacuum for 8 h.

### Characterizations

The morphologies of the as-synthesized materials were characterized by SEM (HITACHI S-4800) and TEM (JEOL-1010) operated at 100 kV. High-resolution TEM was characterized by Cryo-EM (Thermoscientific themis 300). XRD was performed on a Rigaku D/max-2500 diffractometer with Cu Kα radiation (*λ* = 1.5418 Å) at 40 kV and 200 mA. XPS was determined by VG Scientific ESCALab220i-XL spectrometer using Al Ka radiation. FT-IR spectra of the samples were performed on a Bruker Tensor 27 spectrometer. Liquid ^1^H NMR spectra were recorded on Bruker 400 spectrometer. The X-ray absorption spectroscopy experiment was carried out at Beamline 1W2B at Beijing Synchrotron Radiation Facility.

### Computational method

The DFT calculations were performed through DMol3 package^43–45^. The exchange-correlation energy was calculated within the generalized gradient approximation using the function proposed by Perdew and Wang (PW91) with dispersion correction (DFT-D). Structural optimizations were obtained on the basis of the convergence criterion in which SCF tolerance was 1.0 × 10^−6^ Ha, while the convergence tolerances of energy, maximum force, and maximum displacement were 1.0 × 10^−5^ Ha, 2.0 × 10^−3^ Ha Å^−1^, and 5.0 × 10^−3^ Å, correspondingly. A Fermi smearing of 2.0 × 10^−3^ Ha and a cutoff radius of 5.5 Å were used to accelerate convergence. The double numerical plus polarization (DNP) basis sets with effective core potential were employed to express atomic potentials. The adsorption energy between the segment of structural units of Cu-MOF and adsorbed gas (CO_2_) is determined using ∆*E* = *E*(A_n_B_m_) – n*E(A) – m*E(B), where *E*(A_n_B_m_), *E*(A), and *E*(B) denote the total energies of the segment of A_n_B_m_, A, and B, respectively^46,47^.

### Catalysis study

The procedure was similar to that reported by Dhakshinamoorthy and co-workers^38^. Benzyl alcohol 20 mg, catalyst 30 mg, TEMPO 14 mg, Na_2_CO_3_ 20 mg, and 1 mL DMF were added in a 5 mL flask. The reaction temperature was kept at 75 °C and stirred at oxygen atmosphere. After the desired time, the heterogeneous mixture was cooled and centrifuged. The liquid mixture was analyzed by ^1^H-NMR method, which was conducted on a Bruker Avance III 400 HD spectrometer in DMSO-d6. For the reusability study, the N-Cu(BDC) catalyst after 2.5 h of reaction was recovered by centrifugation, washed with ethanol and dried under vacuum. Then the catalyst was added into 1 mL DMF containing TEMPO (14 mg), Na_2_CO_3_ (20 mg) and benzyl alcohol (20 mg) for a consecutive run. ICP-AES (VISTA-MPX) was used to determine whether there was Cu leached to DMF after reaction for five runs.

## Supplementary information

Supplementary Information

## Data Availability

The data Supplementary the findings of this study are available within the article and its Supplementary Information. All data is available from the authors upon reasonable request.

## Code availability

All code supporting the findings of this study are available from the corresponding author on request.

## References

[1] Yaghi OM (2003). Reticular synthesis and the design of new materials. Nature.

[2] Xie K (2018). Continuous assembly of a polymer on a metal-organic framework (CAP on MOF): A 30 nm thick polymeric gas separation membrane. Energy Environ. Sci..

[3] Jeong GY (2018). Metal-organic framework patterns and membranes with heterogeneous pores for flow-assisted switchable separations. Nat. Commun..

[4] Li YP (2019). Ultramicroporous building units as a path to bi-microporous metal-organic frameworks with high acetylene storage and separation performance. Angew. Chem. Int. Ed..

[5] Wang Q (2020). State of the art and prospects in metal-organic framework (MOF)-based and MOF-derived nanocatalysis. Chem. Rev..

[6] Dolgopolova EA (2018). Photochemistry and photophysics of MOFs: steps towards MOF-based sensing enhancements. Chem. Soc. Rev..

[7] Bai Y (2016). Zr-based metal-organic frameworks: design, synthesis, structure, and applications. Chem. Soc. Rev..

[8] Phang WJ (2015). Superprotonic conductivity of a UiO-66 framework functionalized with sulfonic acid groups by facile postsynthetic oxidation. Angew. Chem. Int. Ed..

[9] Zhao MT (2018). Two-dimensional metal-organic framework nanosheets: synthesis and applications. Chem. Soc. Rev..

[10] Peng Y (2014). Metal-organic framework nanosheets as building blocks for molecular sieving membranes. Science.

[11] Zhao SL (2016). Ultrathin metal-organic framework nanosheets for electrocatalytic oxygen evolution. Nat. Energy.

[12] Huang J (2018). Electrochemical exfoliation of pillared-layer metal-organic framework to boost the oxygen evolution reaction. Angew. Chem. Int. Ed..

[13] Wang XR (2017). Reversed thermo-switchable molecular sieving membranes composed of two-dimensional metal-organic nanosheets for gas separation. Nat. Commun..

[14] Rodenas T (2010). Metal-organic framework nanosheets in polymer composite materials for gas separation. Nat. Mater..

[15] Dhakshinamoorth A, Asiri AM, Garcia H (2019). 2D metal-organic frameworks as multifunctional materials in heterogeneous catalysis and electro/photocatalysis. Adv. Mater..

[16] Mukhopadhyay A (2018). Photochromic 2D metal-organic framework nanosheets (MONs): design, synthesis, and functional MON-Ormosil composite. Chem.

[17] Liao WM (2018). Tailoring exciton and excimer emission in an exfoliated ultrathin 2D metal-organic framework. Nat. Commun..

[18] Sakamoto R (2016). The coordination nanosheet (CONASH). Coord. Chem. Rev..

[19] Ding YJ (2017). Controlled intercalation and chemical exfoliation of layered metal-organic frameworks using a chemically labile intercalating agent. J. Am. Chem. Soc..

[20] Zhao MT (2015). Ultrathin 2D metal-organic framework nanosheets. Adv. Mater..

[21] Choi M (2009). Stable single-unit-cell nanosheets of zeolite MFI as active and long-lived catalysts. Nature.

[22] Peng L (2012). Surfactant-directed assembly of mesoporous metal-organic framework nanoplates in ionic liquids. Chem. Commun..

[23] Zhang X (2019). Fabrication of 2D metal-organic framework nanosheets with tailorable thickness using bio-based surfactants and their application in catalysis. Green Chem..

[24] Liu M (2018). Ultrathin metal-organic framework nanosheets as a gutter layer for flexible composite gas separation membranes. ACS Nano.

[25] Zuo Q (2019). Ultrathin metal-organic framework nanosheets with ultrahigh loading of single Pt atoms for efficient visible-light-driven photocatalytic H_2_ evolution. Angew. Chem. Int. Ed..

[26] Zhan G (2019). Fabrication of ultrathin 2D Cu-BDC nanosheets and the derived integrated MOF nanocomposites. Adv. Funct. Mater..

[27] Carson CG (2009). Synthesis and structure characterization of copper terephthalate metal-organic frameworks. Eur. J. Inorg. Chem..

[28] Trześniewski BJ (2015). In situ observation of active oxygen species in Fe-containing Ni based oxygen evolution catalysts: The effect of pH on electrochemical activity. J. Am. Chem. Soc..

[29] Yang HB (2018). Atomically dispersed Ni(I) as the active site for electrochemical CO_2_ reduction. Nat. Energy.

[30] Huang JH (2015). CoOOH nanosheets with high mass activity for water oxidation. Angew. Chem. Int. Ed..

[31] Liu CC (2016). Metal-organic framework for emulsifying carbon dioxide and water. Angew. Chem. Int. Ed..

[32] Tsuruoka T (2009). Nanoporous nanorods fabricated by coordination modulation and oriented attachment growth. Angew. Chem. Int. Ed..

[33] Pham M-H, Vuong G-T, Fontaine F-G, Do T-O (2012). A route to bimodal micro-mesoporous metal-organic frameworks nanocrystals. Cryst. Growth Des..

[34] Peng L (2014). Highly mesoporous metal-organic framework assembled in a switchable solvent. Nat. Commun..

[35] Zakzeski J (2010). The catalytic valorization of lignin for the production of renewable chemicals. Chem. Rev..

[36] Rahimi A (2013). Chemoselective metal-free aerobic alcohol oxidation in lignin. J. Am. Chem. Soc..

[37] Corma A (2008). Supported gold nanoparticles as catalysts for organic reactions. Chem. Soc. Rev..

[38] Dhakshinamoorthy A (2011). Aerobic oxidation of benzylic alcohols catalyzed by metal-organic frameworks assisted by TEMPO. ACS Catal..

[39] Sang XX (2017). Ionic liquid accelerates the crystallization of Zr-based metal-organic frameworks. Nat. Commun..

[40] Zhao MT (2017). Two-dimensional metal-organic framework nanosheets. Small Methods.

[41] Makiura R (2010). Surface nano-architecture of a metal-organic framework. Nat. Mater..

[42] Zhuang LZ (2019). A surfactant-free and scalable general strategy for synthesizing ultrathin two-dimensional metal-organic framework nanosheets for the oxygen evolution reaction. Angew. Chem. Int. Ed..

[43] Delley B (2000). From molecules to solids with the DMol3 approach. J. Chem. Phys..

[44] Delley B (1996). Fast calculation of electrostatics in crystals and large molecules. J. Phys. Chem..

[45] Delley B (1990). An all-electron numerical method for solving the local density functional for polyatomic molecules. J. Chem. Phys..

[46] Hong XJ (2018). Confinement of polysulfides within bi-functional metal-organic frameworks for high performance lithium-sulfur batteries. Nanoscale.

[47] Halder S (2017). A Ni-based MOF for selective detection and removal of Hg^2+^ in aqueous medium: a facile strategy. Dalton Trans..

