# Supplementary material for: CO2 controls the oriented growth of metal-organic framework with highly accessible active sites
Source: Nat Commun. 2020 Mar 18;11:1431. doi: 10.1038/s41467-020-15200-4 (PMC7080726; doi:10.1038/s41467-020-15200-4)
Supplement: Supplementary file 1 — Supplementary Information [file 41467_2020_15200_MOESM1_ESM.pdf]

CO_2 Controls the Oriented Growth of Metal-Organic Framework with Highly Accessible Active Sites

Zhang et al.

Supplementary Figures

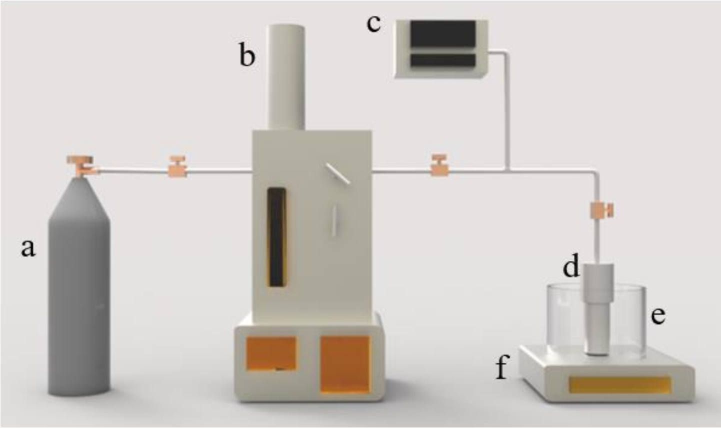

Supplementary Figure 1 | Diagram of the device used for MOF synthesis. a gas cylinder; b high-pressure pump; c pressure gage; d autoclave; e water bath; f magnetic stirring apparatus. The apparatus used for synthesizing N-Cu(BDC) was similar to that used previously.[1,2] In a typical experiment, the desired amount of $Cu(NO_3)_2{\cdot}3H_2O$, H_2BDC, TEA and methanol were added into the autoclave. The autoclave was put in a water bath, and then CO_2 was charged into the autoclave to the desired pressure. The mixture was stirred for 24 h, then the stirrer was stopped. After releasing CO_2, the precipitate was collected, washed and dried.

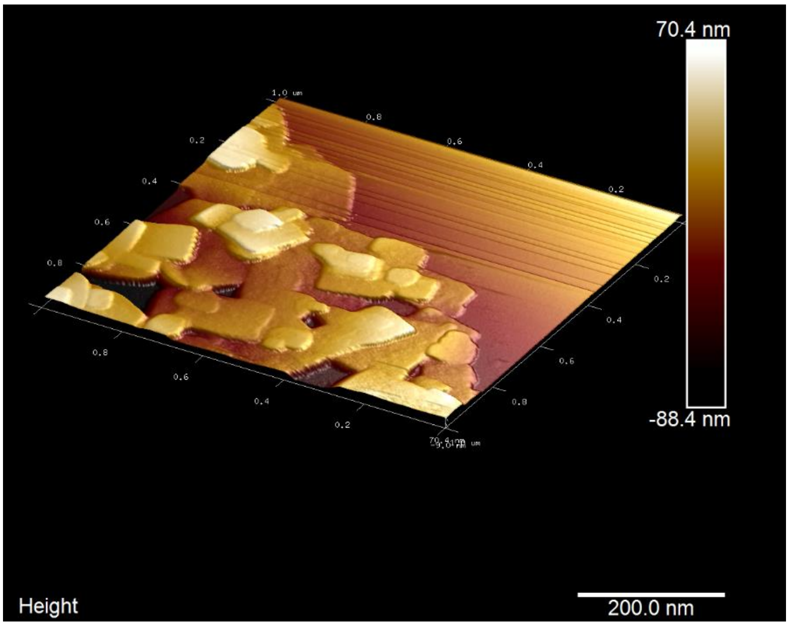

Supplementary Figure 2 | 3D AFM image of N-Cu(BDC) synthesized in CO_2/methanol solution at 7.38 MPa and 35 ^oC with 0.1 mL of TEA for 24 h.

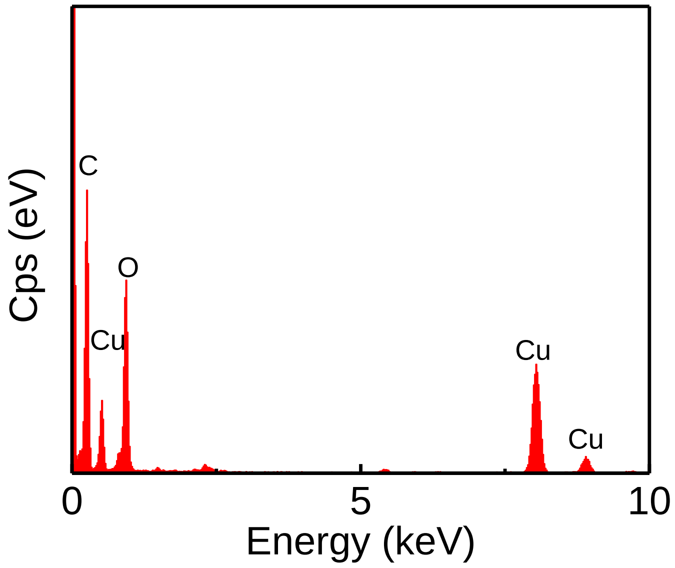

Supplementary Figure 3 | EDS spectrum of N-Cu(BDC) synthesized in CO_2/methanol solution at 7.38 MPa and 35 °C with 0.1 mL of TEA for 24 h.

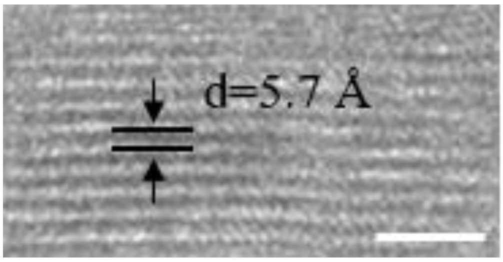

Supplementary Figure 4 | High-resolution TEM image of N-Cu(BDC) synthesized in CO_2/methanol solution at 7.38 MPa and 35 ^oC with 0.1 mL of TEA for 24 h. Scale bar: 3 nm.

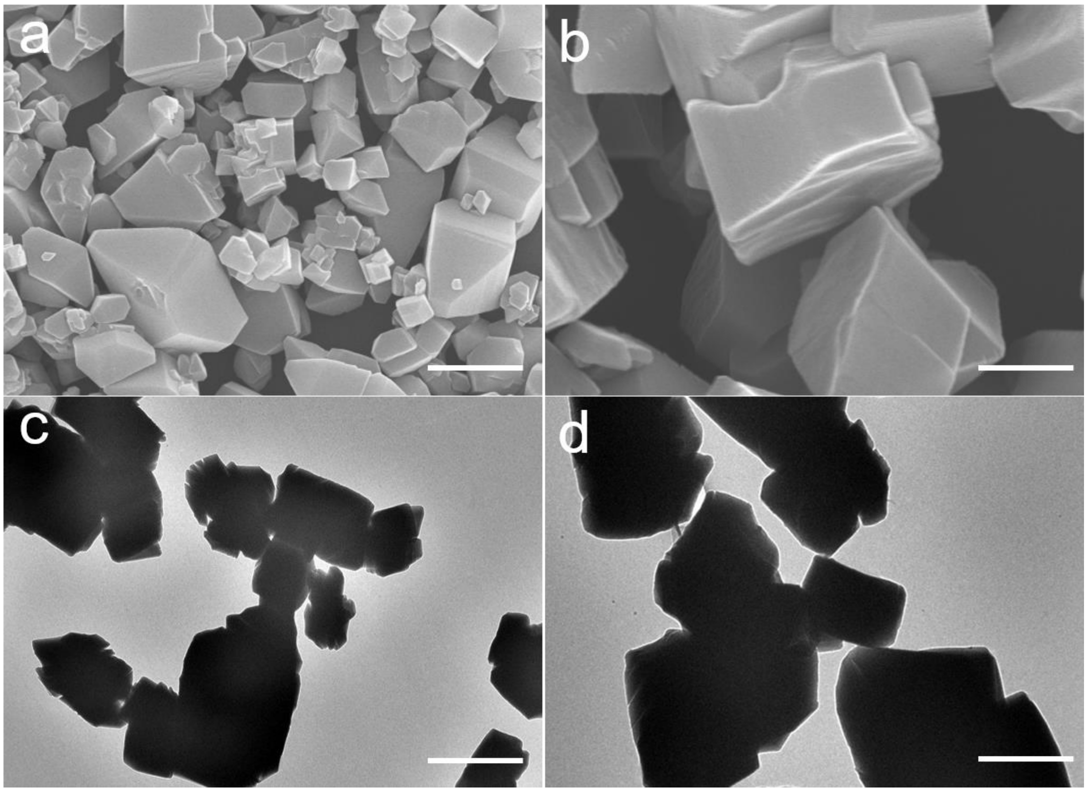

Supplementary Figure 5 | Characterizations of B-Cu(BDC). SEM (a, b) and TEM (c, d) images of B-Cu(BDC) synthesized by hydrothermal method. Scale bars: 2 μm in a, 400 nm in b, 1 μm in c and 500 nm in d.

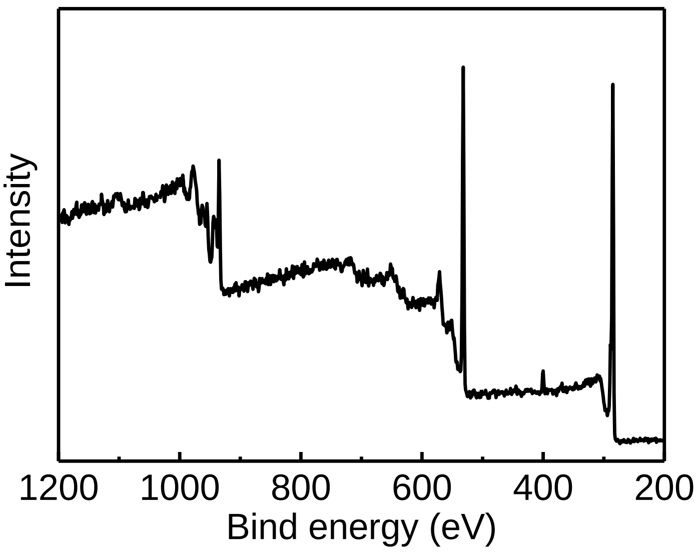

Supplementary Figure 6 | Wide-range XPS spectrum of N-Cu(BDC) synthesized in CO_2/methanol solution at 7.38 MPa and 35 ^{o}C with 0.1 mL of TEA for 24 h.

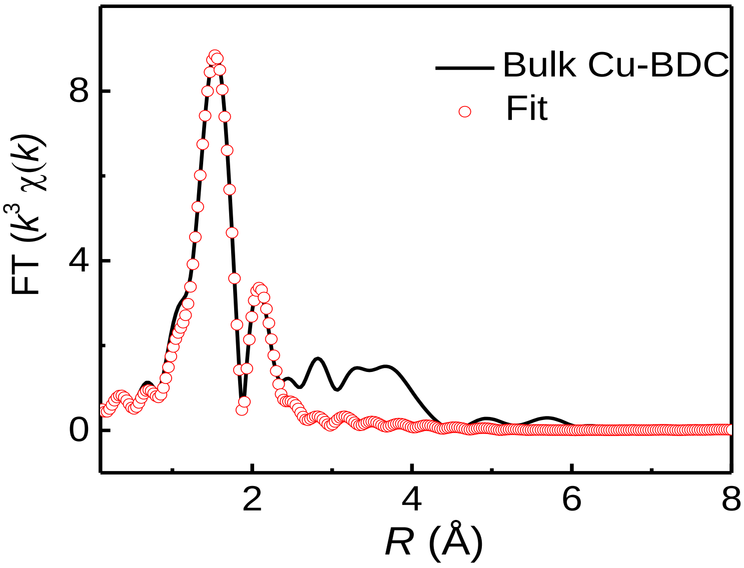

Supplementary Figure 7 | EXAFS fitting curve of B-Cu(BDC).

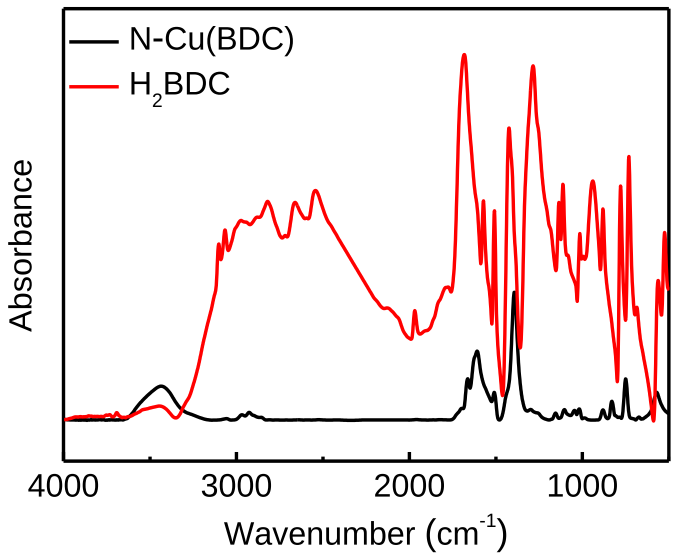

Supplementary Figure 8 | FT-IR spectra of N-Cu(BDC) synthesized in CO_2/methanol solution at 7.38 MPa and 35 °C with 0.1 mL of TEA for 24 h and H_2BDC.

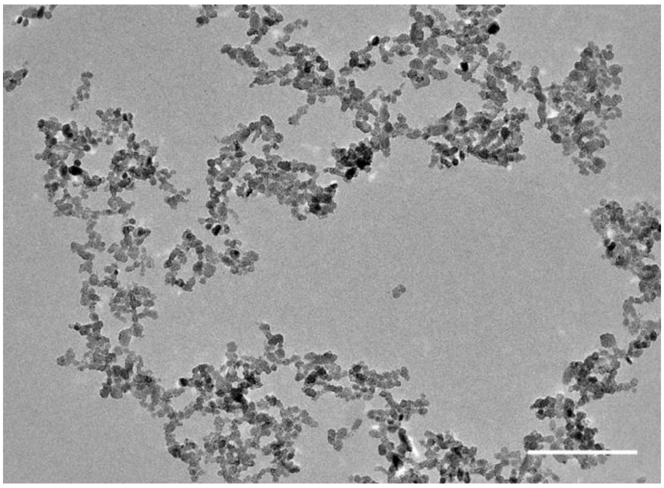

Supplementary Figure 9 | TEM image of Cu(BDC) synthesized in CO_2/methanol solution at 7.38 MPa and 35 ^oC with 0.1 mL of TEA with time of 20 min. Scale bar: 200 nm.

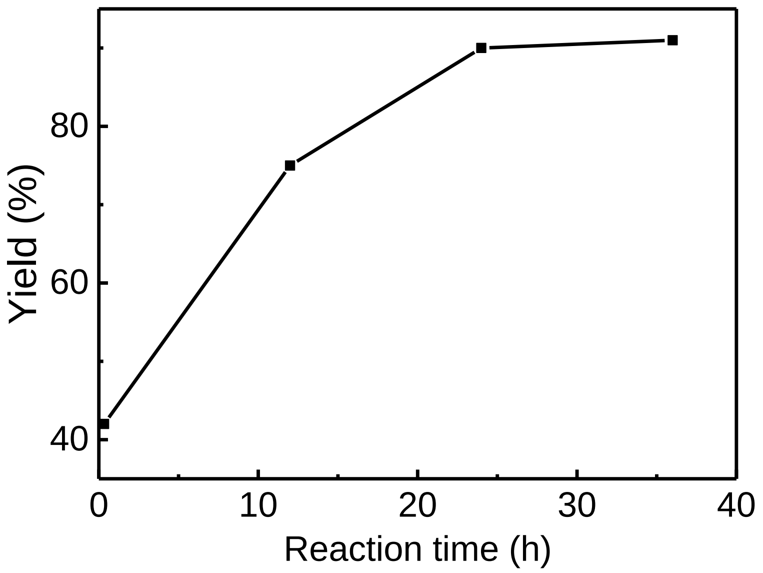

Supplementary Figure 10 | Dependence of yield of Cu(BDC) synthesized at pressure of 7.38 MPa on reaction time.

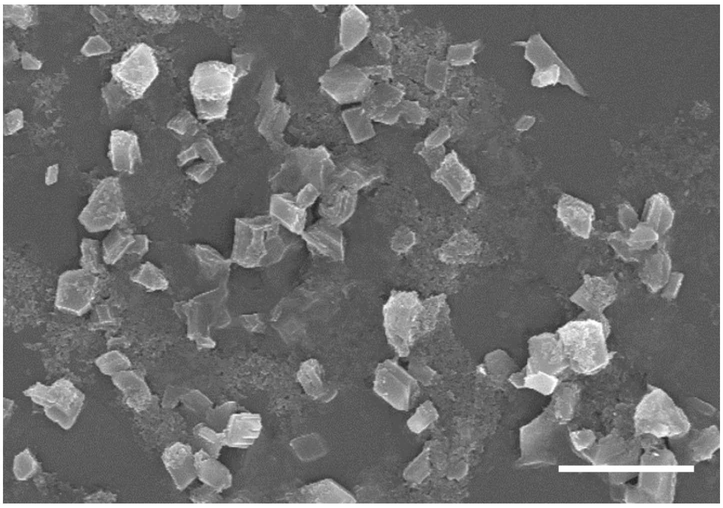

Supplementary Figure 11 | SEM image of the product synthesized in pure methanol at 35 °C with 0.1 mL of TEA for 24 h. Scale bar: 2 μm.

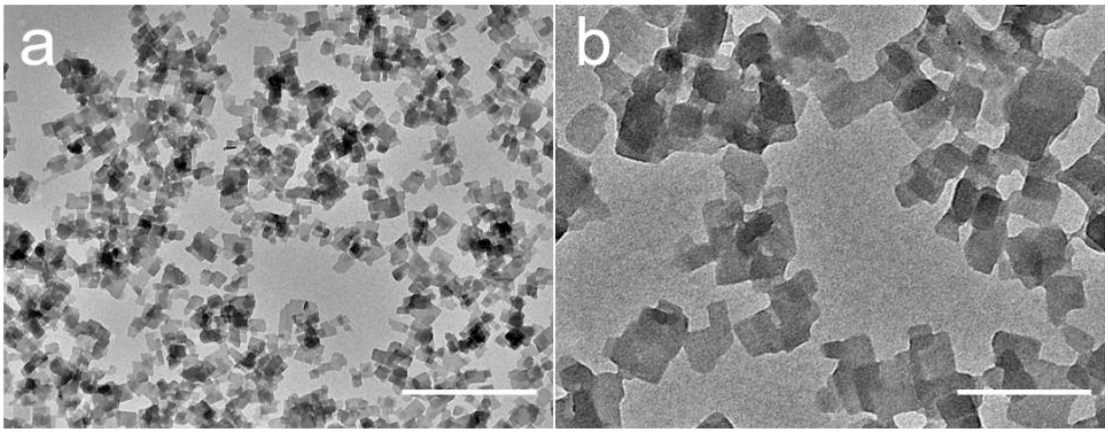

Supplementary Figure 12 | TEM images of the N-Cu(BDC) synthesized in CO_2/methanol solution at 10.50 MPa and 35 ^{o}C with 0.1 mL of TEA for 24 h. Scale bars: 1 μm in a and 300 nm in b.

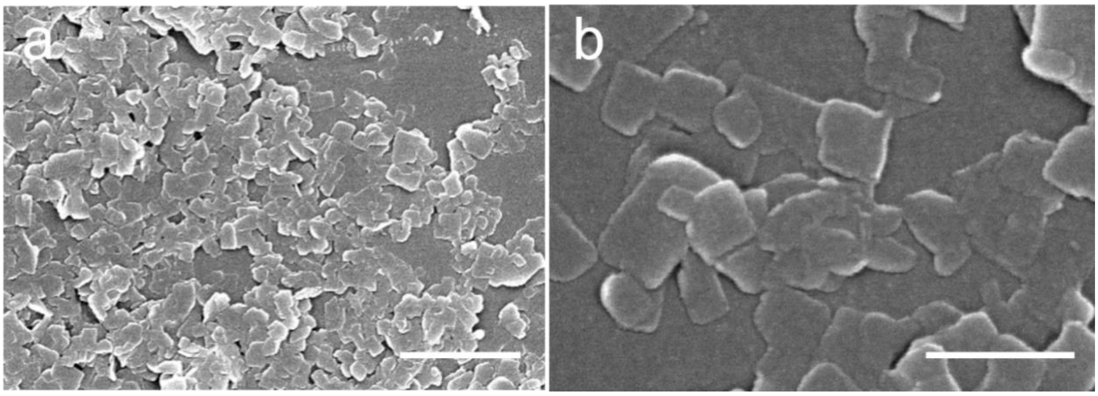

Supplementary Figure 13 | SEM images of N-Cu(BDC) synthesized in CO_2/methanol solution at 8.60 MPa and 35 ^oC with 0.1 mL of TEA for 24 h. Scale bars: 1 μm in a and 300 nm in b.

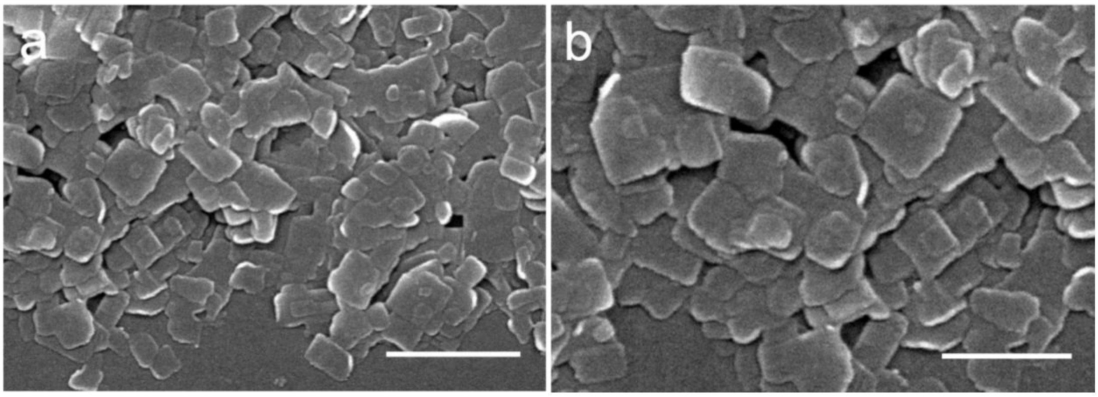

Supplementary Figure 14 | SEM images of N-Cu(BDC) synthesized in CO_2/methanol solution at 10.50 MPa and 35 °C with 0.1 mL of TEA for 24 h. Scale bars: 500 nm in a and 300 nm in b.

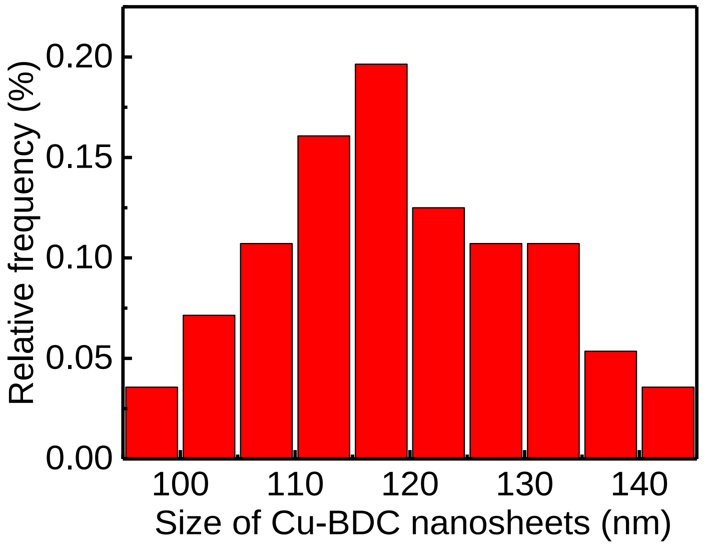

Supplementary Figure 15 | Size distribution of N-Cu(BDC) synthesized in CO_2/methanol solution at 7.38 MPa and 35 °C with 0.1 mL of TEA for 24 h.

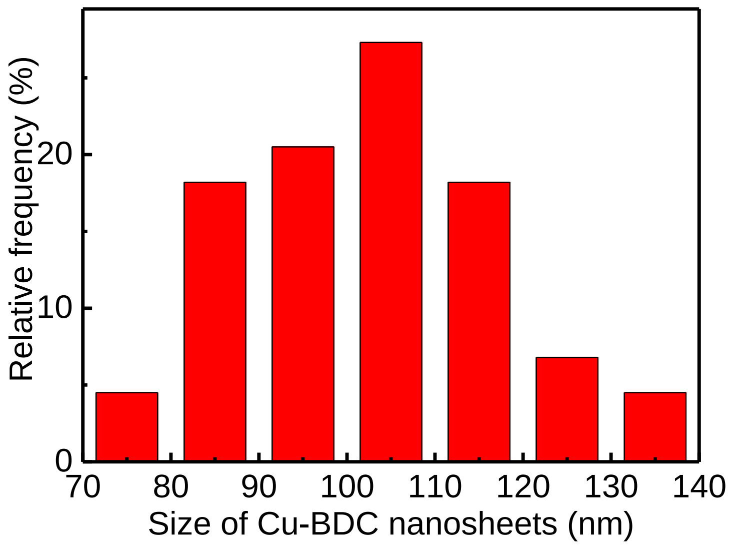

Supplementary Figure 16 | Size distribution of N-Cu(BDC) synthesized in CO_2/methanol solution at 8.60 MPa and 35 °C with 0.1 mL of TEA for 24 h.

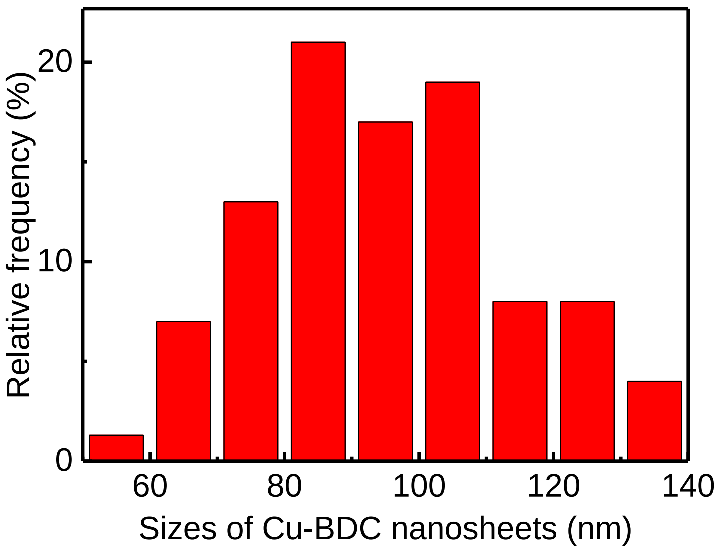

Supplementary Figure 17 | Size distribution of N-Cu(BDC) synthesized in CO_2/methanol solution at 10.50 MPa and 35 °C with 0.1 mL of TEA for 24 h.

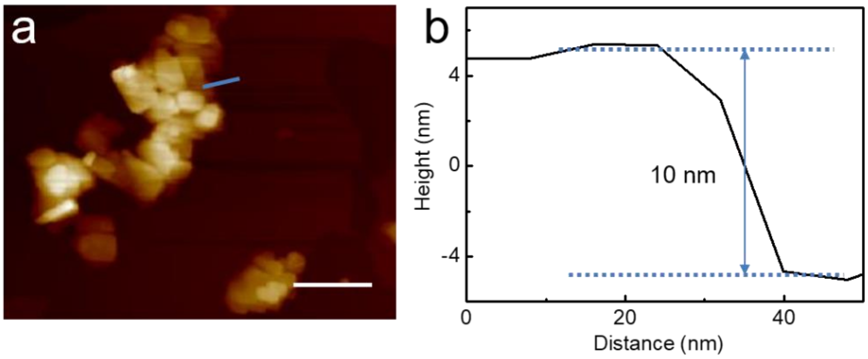

Supplementary Figure 18 | AFM image (a) and corresponding height profile (b) of N-Cu(BDC) synthesized in CO_2/methanol solution at 8.60 MPa and 35 ^oC with 0.1 mL of TEA for 24 h. Scale bar: 500 nm in a.

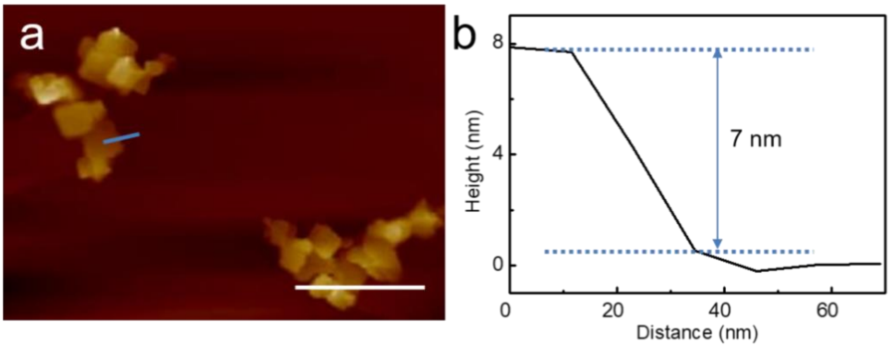

Supplementary Figure 19 | AFM image (a) and corresponding height profile (b) of N-Cu(BDC) synthesized in CO_2/methanol solution at 10.50 MPa and 35 ^oC with 0.1 mL of TEA for 24 h. Scale bar: 500 nm in a.

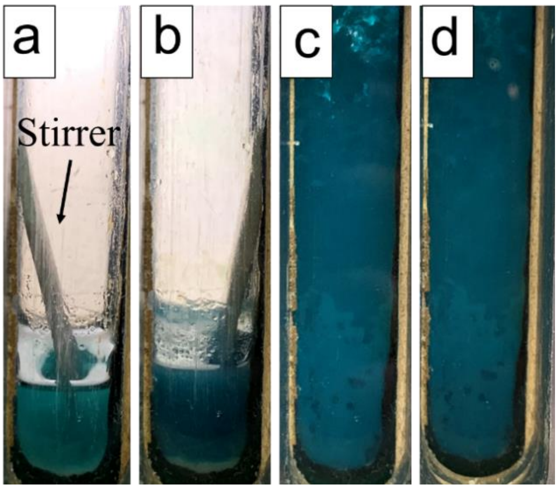

Supplementary Figure 20 | Photographs of Cu(BDC) synthesis in methanol (a) and CO_2/methanol solution at 3.40 MPa (b), 6.40 MPa (c) and 7.38 MPa (d). The temperature was 35 °C. The apparatus and experimental procedures were similar to those reported previously[3]. It consisted mainly of a high-pressure view cell of 50 mL, a constant temperature water bath, a high-pressure syringe pump (DB-80), a pressure gauge, a magnetic stirrer and a gas cylinder. The temperature of the water bath was controlled by a HAAKE D8 digital controller. In a typical experiment, the desired amount of $Cu(NO_3)_2 \cdot 3H_2O$, H_2BDC, TEA and methanol were added into cell, and the cell was placed in the constant temperature water bath (35 °C). Then a suitable amount of CO_2 was charged into the high-pressure cell under stirring.

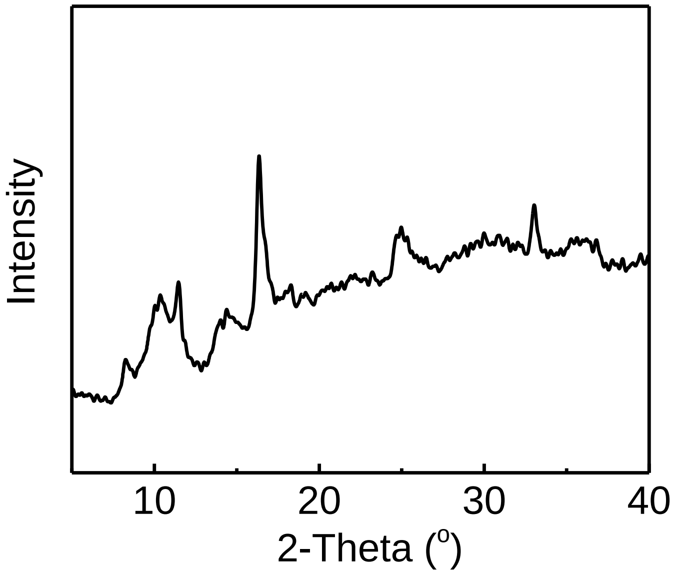

Supplementary Figure 21 | XRD pattern of the Cu(BDC) synthesized in CO_2/methanol solution at 7.38 MPa and 25 ^{o}C with 0.1 mL of TEA for 24 h.

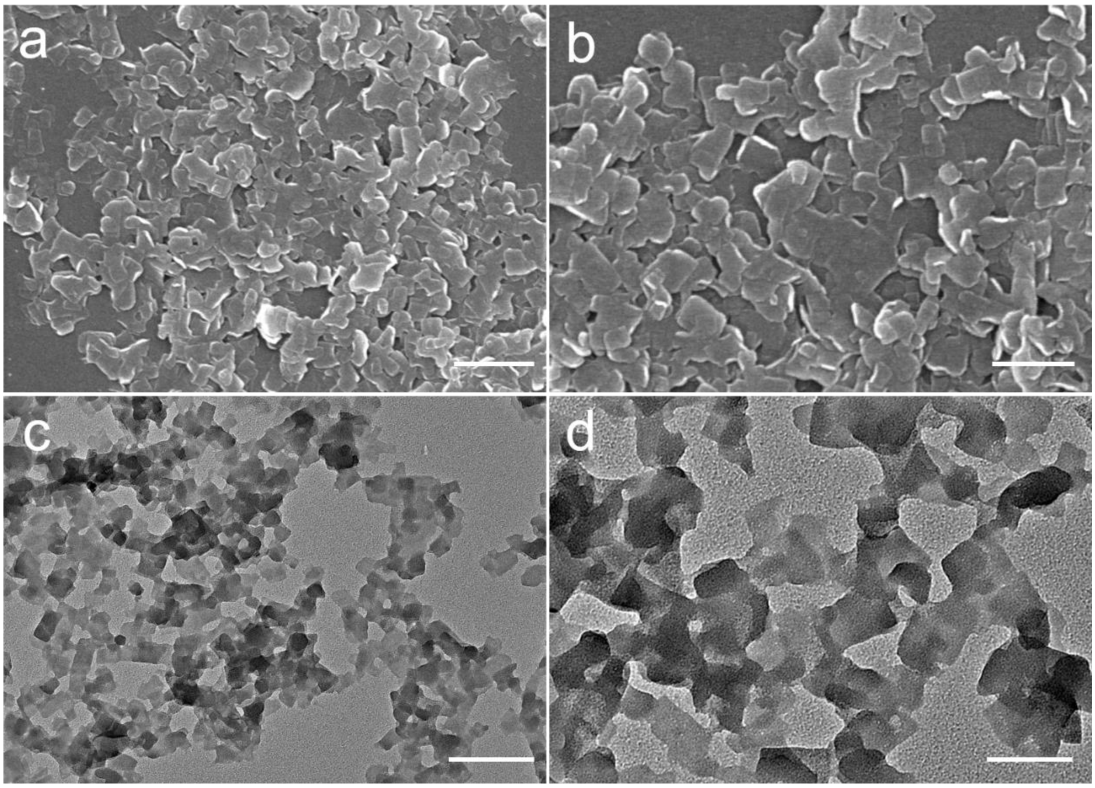

Supplementary Figure 22 | SEM (a, b) and TEM (c, d) images of the Cu(BDC) synthesized in CO_2/methanol solution at 7.38 MPa and 25 ^oC with 0.1 mL of TEA for 24 h. Scale bars: 500 nm in a, 300 nm in b, 400 nm in c and 200 nm in d.

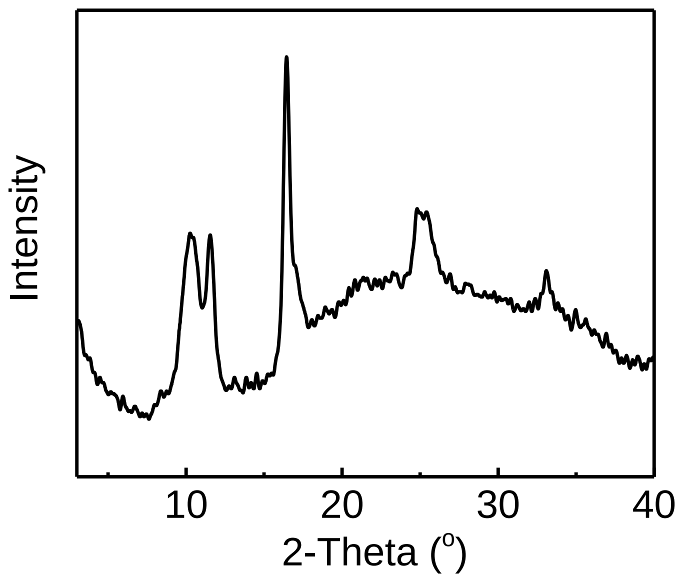

Supplementary Figure 23 | XRD pattern of Cu(BDC) synthesized in CO_2/methanol solution at 7.38 MPa and 45 °C with 0.1 mL of TEA for 24 h.

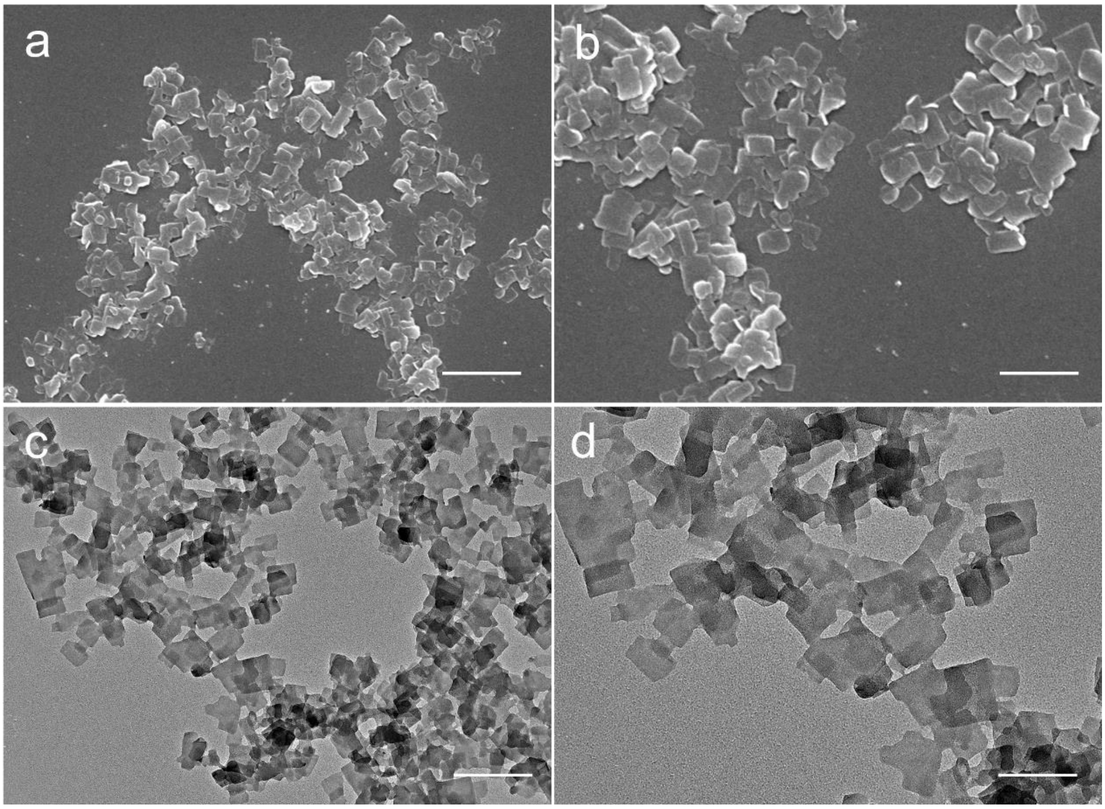

Supplementary Figure 24 | SEM (a, b) and TEM (c, d) images of Cu(BDC) synthesized in CO_2/methanol solution at 7.38 MPa and 45 ^oC with 0.1 mL of TEA for 24 h. Scale bars: 1 μm in a, 500 nm in b and c and 300 nm in d.

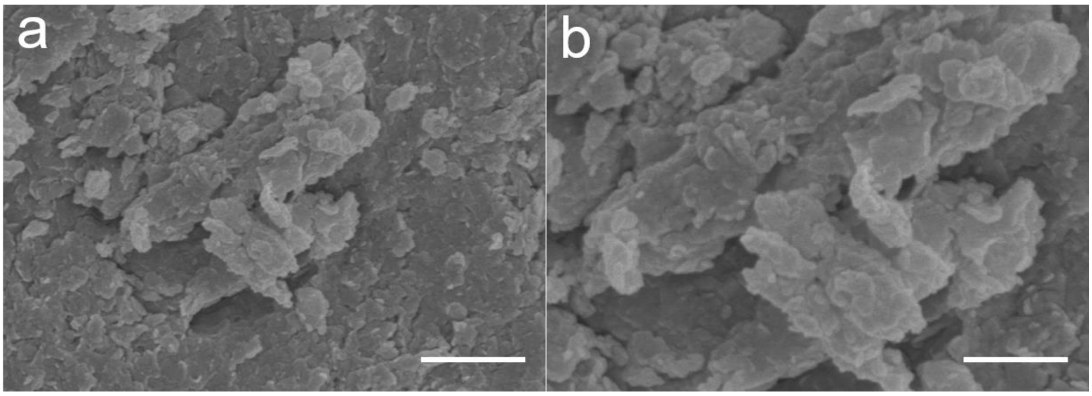

Supplementary Figure 25 | SEM images of the product synthesized in N_2/ methanol solution at 7.30 MPa and 35 ^oC with 0.1 mL of TEA for 24 h. Scale bars: 1 μm in a and 500 nm in b.

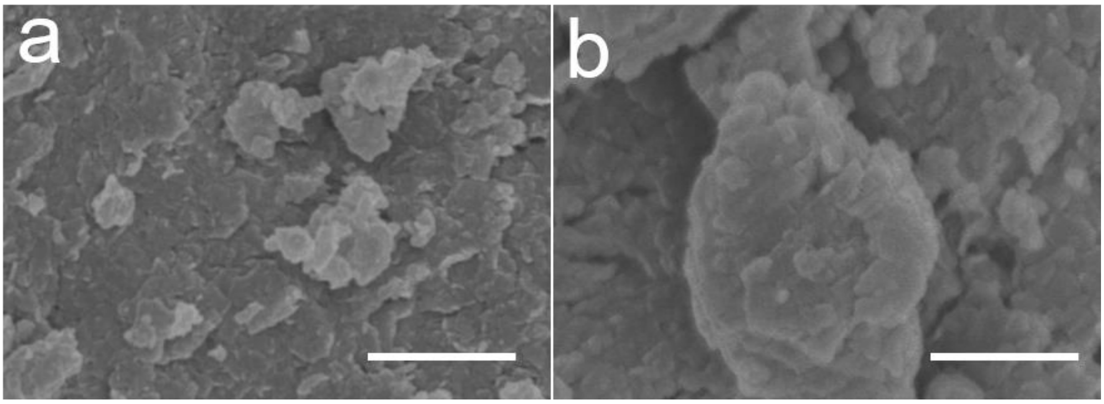

Supplementary Figure 26 | SEM images of the product synthesized in Ar/ methanol solution at 7.30 MPa and 35 °C with 0.1 mL of TEA for 24 h. Scale bars: 2 μm in a and 500 nm in b.

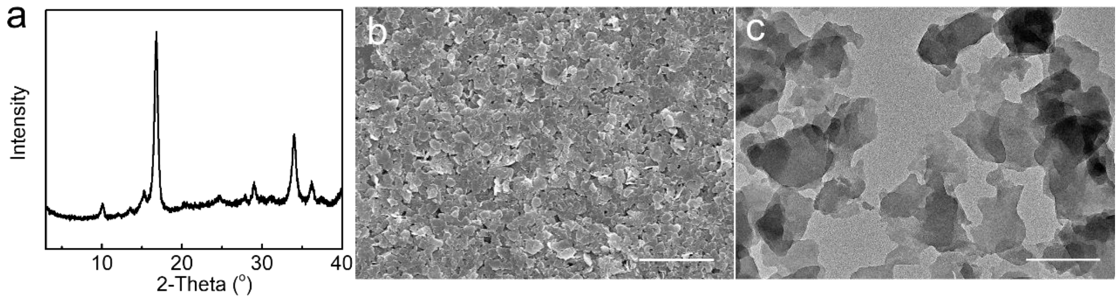

Supplementary Figure 27 | XRD pattern (a), SEM (b) and TEM (c) images of the Cu(BDC) synthesized in CO_2/acetonitrile solution at 7.38 MPa and 35 ^{o}C with 0.1 mL of TEA for 24 h. Scale bars: 3 μm in b and 400 nm in c.

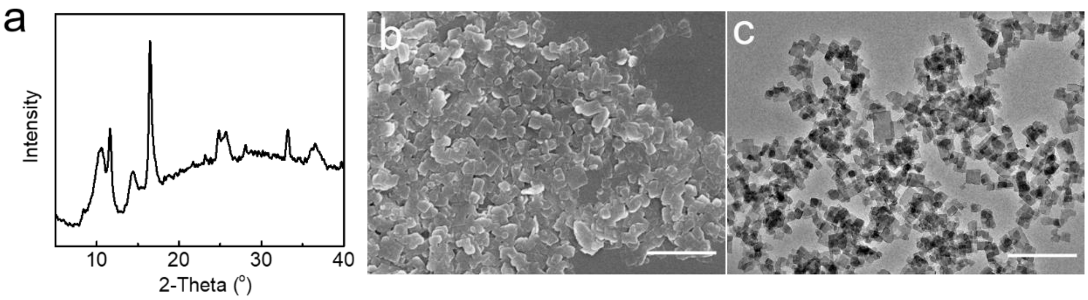

Supplementary Figure 28 | XRD pattern (a), SEM (b) and TEM (c) images of the Cu(BDC) synthesized in CO_2/ethanol solution at 7.38 MPa and 35 ^oC with 0.1 mL of TEA for 24 h. Scale bar: 500 nm in b and c.

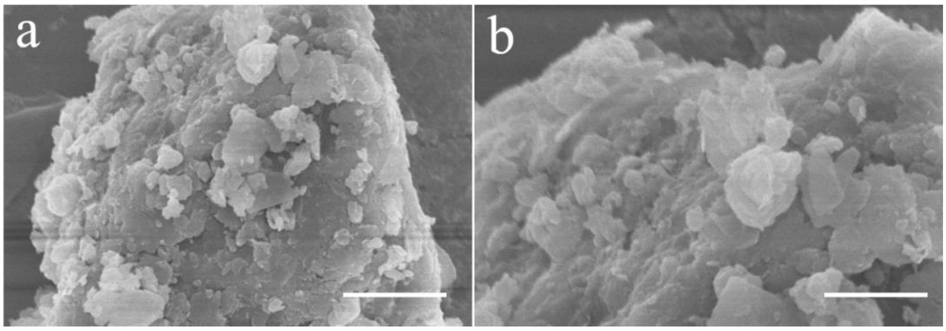

Supplementary Figure 29 | SEM images of the product synthesized in Na_2CO_3/methanol solution at 35 ^oC with 0.1 mL of TEA for 24 h. Scale bars: 2 μm in a and 1 μm in b.

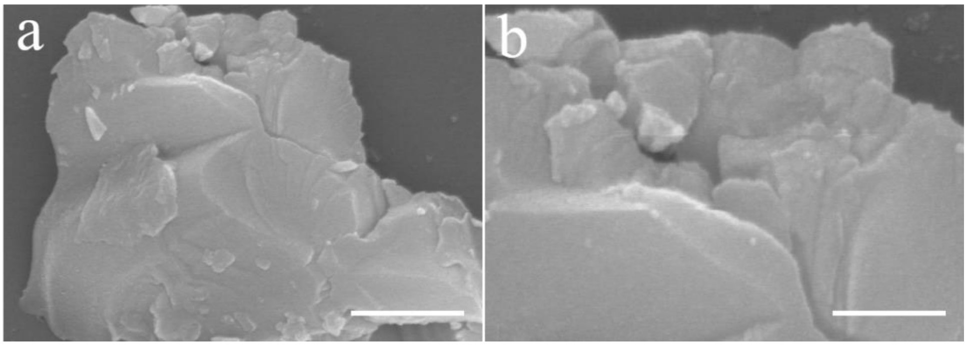

Supplementary Figure 30 | SEM images of the product synthesized in $NaHCO_3$/methanol solution at 35 °C with 0.1 mL of TEA for 24 h. Scale bars: 500 nm in a and 200 nm in b.

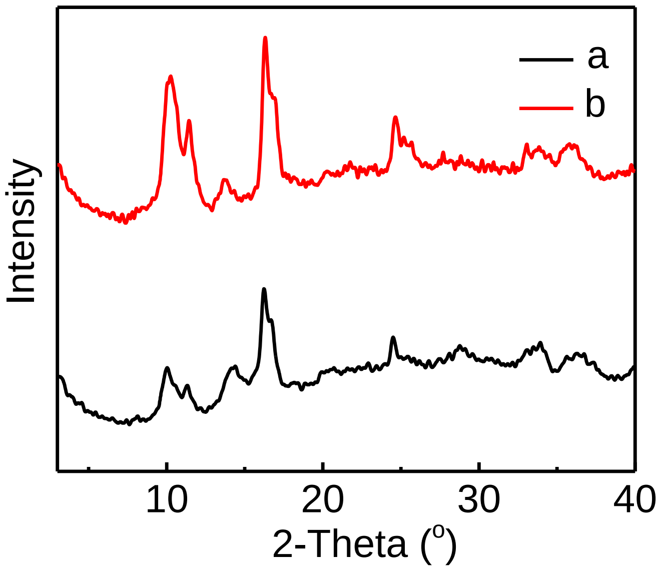

Supplementary Figure 31 | XRD patterns of Cu(BDC) synthesized in CO_2/methanol solution at 7.38 MPa and 35 °C with TEA dosage of 50 (a) and 200 µL (b) for 24 h. The product yields by using 50 and 200 µL TEA are 40% and 93%, respectively.

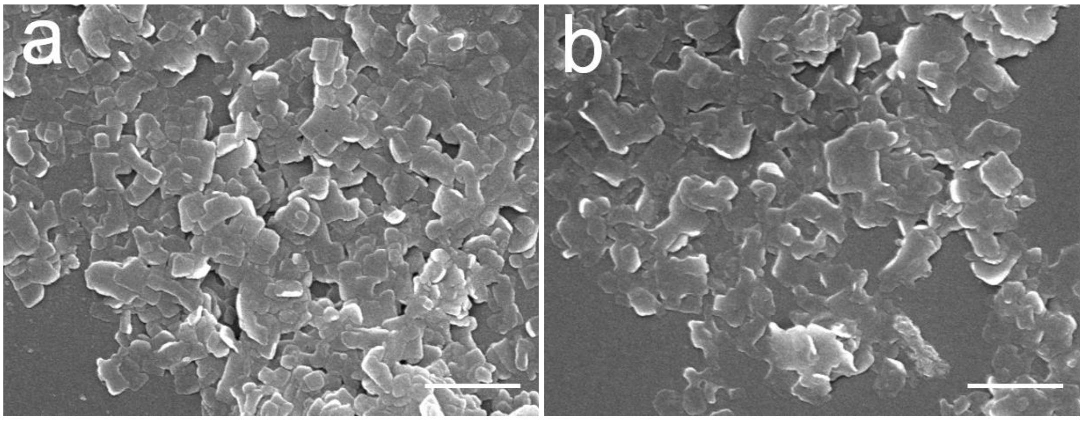

Supplementary Figure 32 | SEM images of the Cu(BDC) synthesized in CO_2/methanol solution at 7.38 MPa and 35 ^{o}C with TEA dosage of 50 (a) and 200 µL (b) for 24 h. Scale bar: 500 nm in a and b.

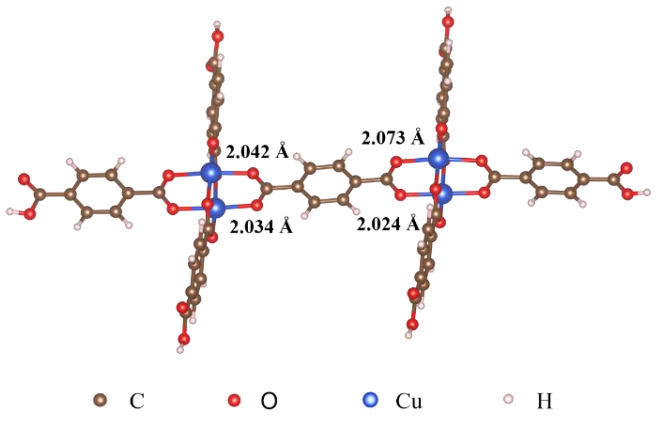

Supplementary Figure 33 | Structural unit of Cu(BDC) and organic ligand along (20-1) plane.

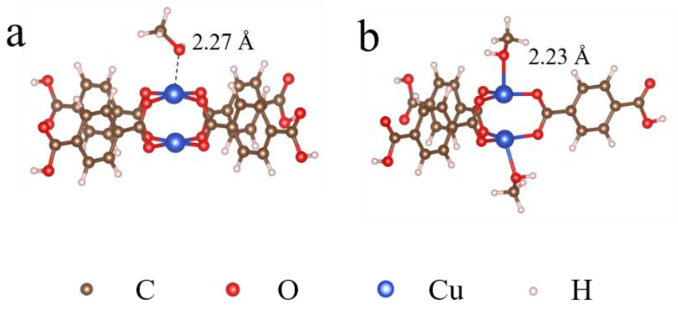

Supplementary Figure 34 | Structural unit of Cu(BDC) and methanol along [20-1] axis (a) and (20-1) plane (b). Although the interactions of Cu(BDC) with methanol are stronger than those of Cu(BDC) with CO_2, CO_2 plays a dominant role at higher pressures because the amount of CO_2 in CO_2/methanol solution is extremely higher than methanol due to the massive dissolution of CO_2 in methanol.

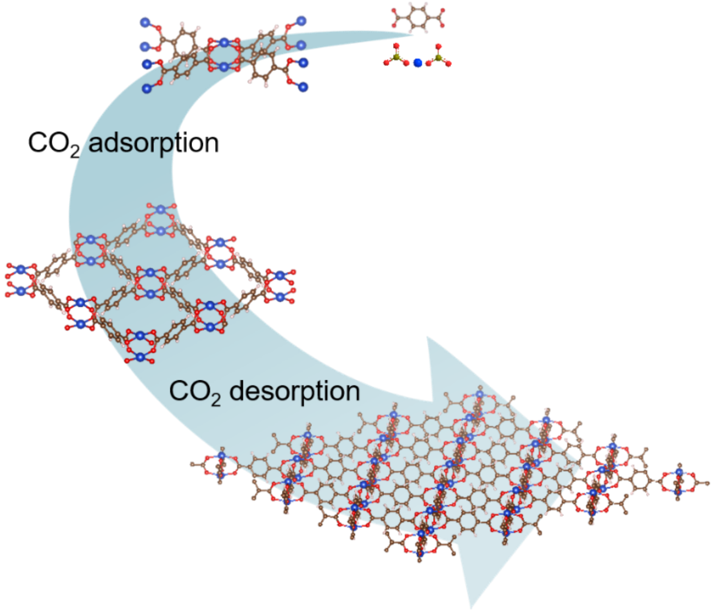

Supplementary Figure 35 | Diagram illustrating the CO_2-directed formation of Cu(BDC) nanosheets.

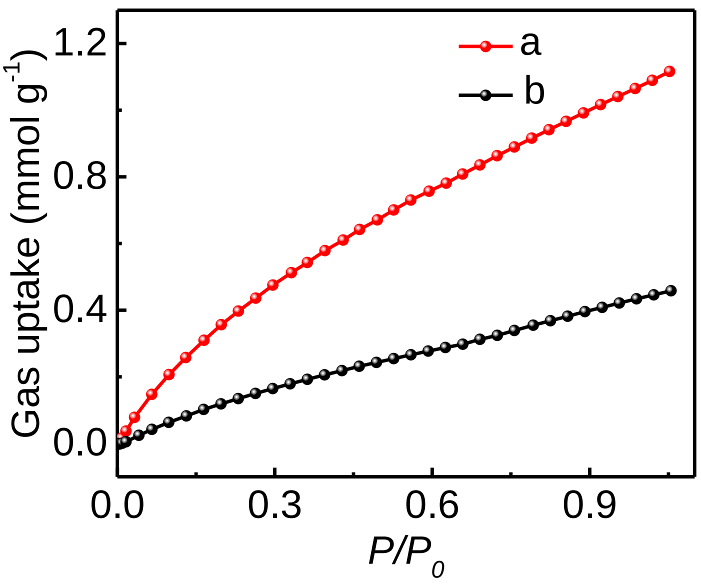

Supplementary Figure 36 | CO_2 adsorption isotherms for N-Cu(BDC) synthesized in CO_2/methanol solution at 7.38 MPa and 35 °C with 0.1 mL of TEA for 24 h (a) and Cu(BDC) synthesized in pure methanol (b).

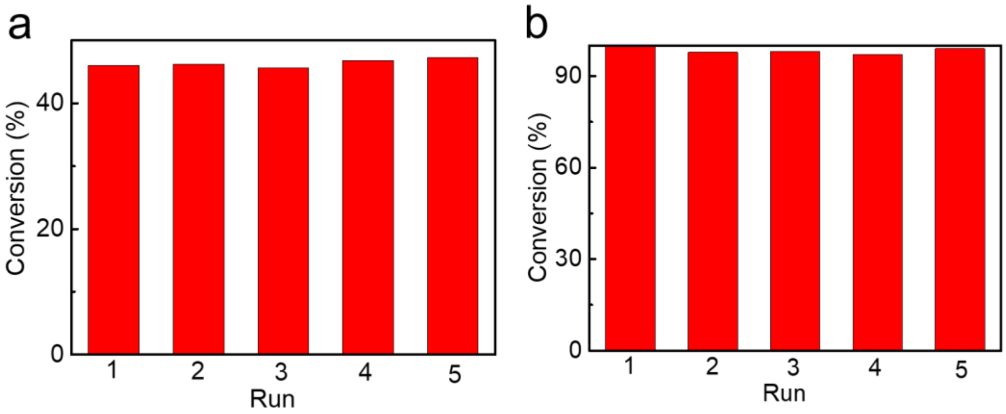

Supplementary Figure 37 | Reusability of N-Cu(BDC) for catalyzing the oxidation of benzyl alcohol to benzaldehyde at reaction time of 0.5 h (a) and 2.5 h (b).

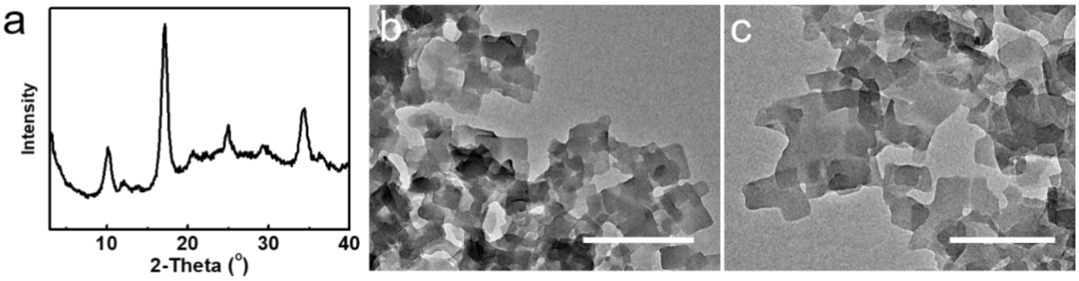

Supplementary Figure 38 | XRD pattern and TEM images of N-Cu(BDC) after 5 catalysis runs. Scale bars: 500 nm in a and 300 nm in b.

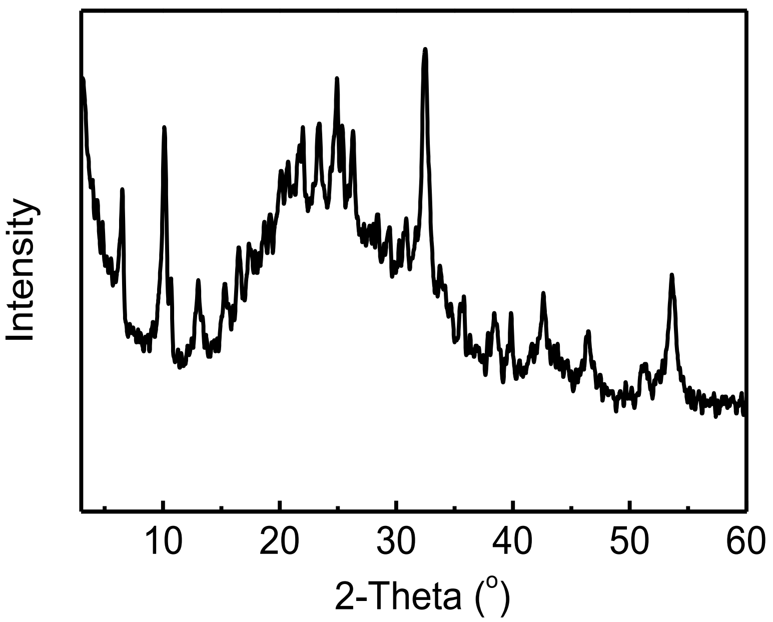

Supplementary Figure 39 | XRD pattern of Co(BDC) nanosheets.

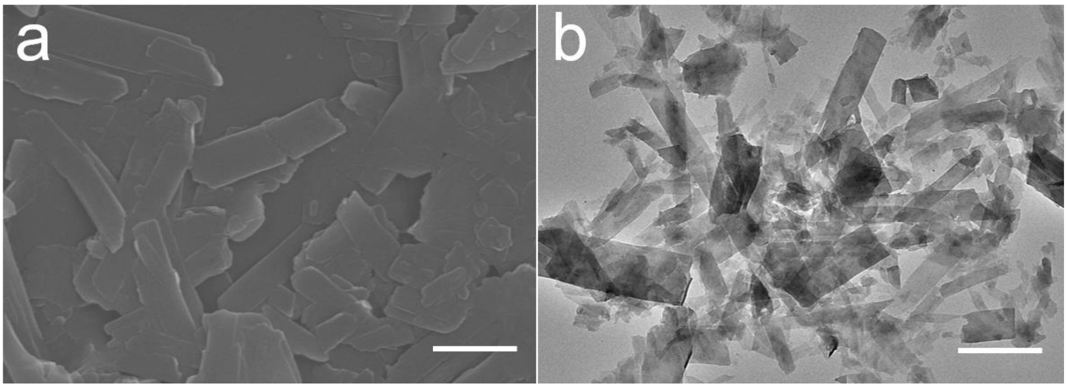

Supplementary Figure 40 | SEM (a) and TEM (b) images of Co(BDC) nanosheets.

Scale bar: 500 nm in a and b.

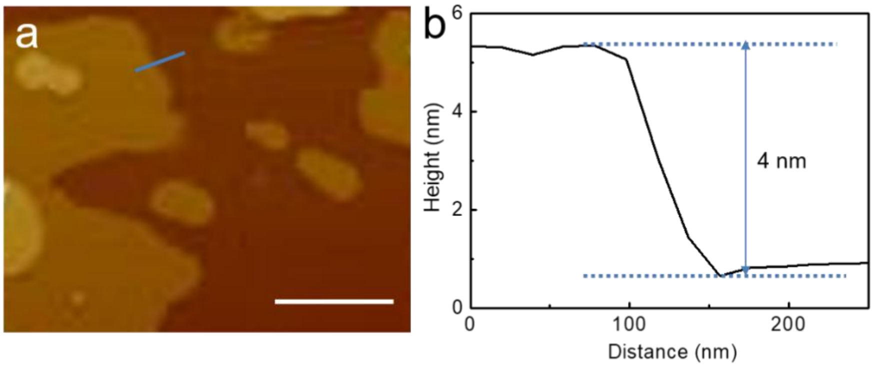

Supplementary Figure 41 | AFM image (a) and corresponding height profile (b) of Co(BDC) nanosheets. Scale bar: 500 nm in a.

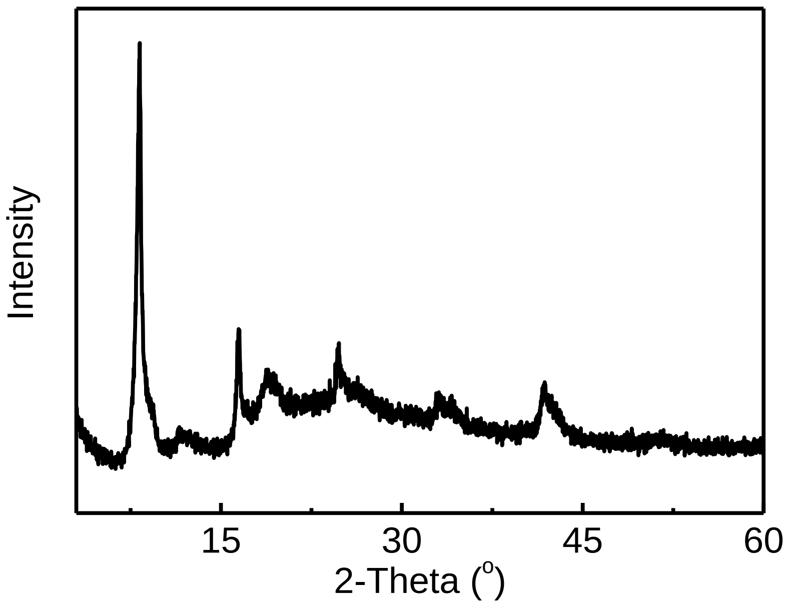

Supplementary Figure 42 | XRD pattern of Cu(1,4-NDC) nanosheets.

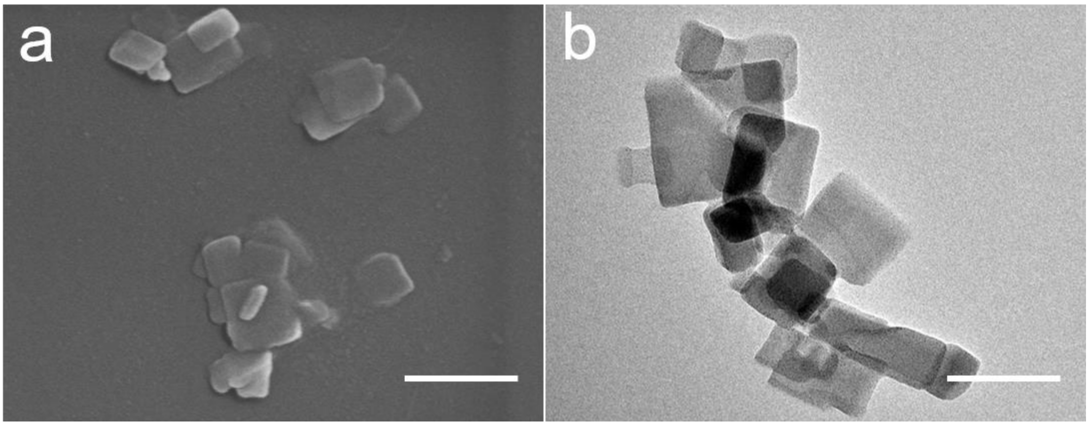

Supplementary Figure 43 | SEM (a) and TEM (b) images of Cu(1,4-NDC) nanosheets. Scale bar: 500 nm in a and b.

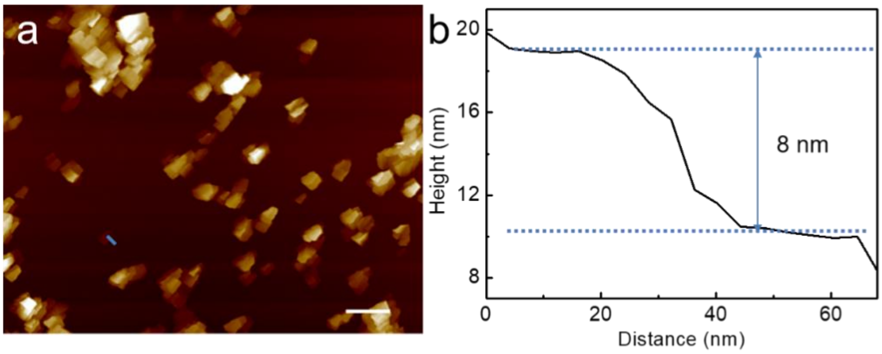

Supplementary Figure 44 | AFM image (a) and corresponding height profile (b) of Cu(1,4-NDC) nanosheets. Scale bar: 1 μm.

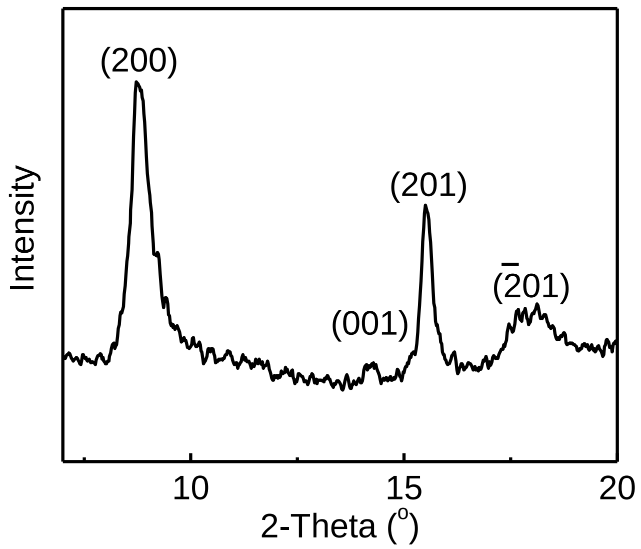

Supplementary Figure 45 | XRD pattern of (Co, Ni)(BDC) nanosheets.

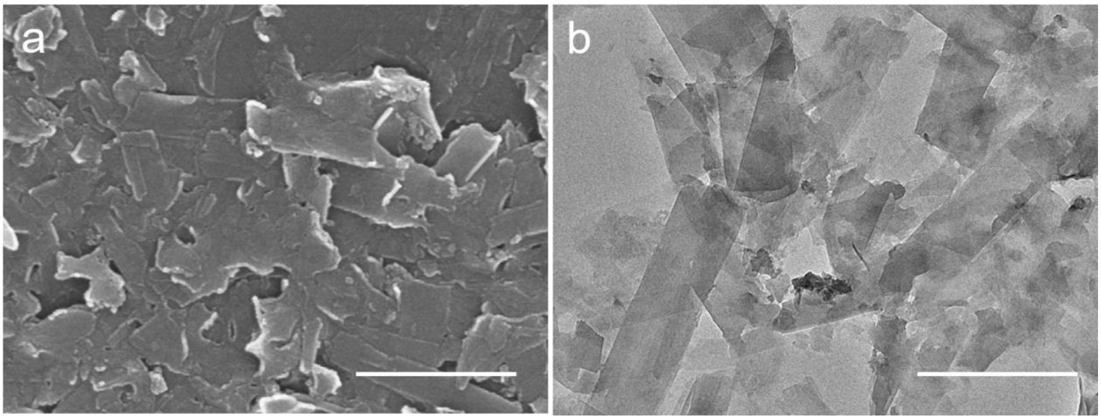

Supplementary Figure 46 | SEM and TEM images of (Co, Ni)(BDC) nanosheets.

Scale bar: 500 nm in a and b.

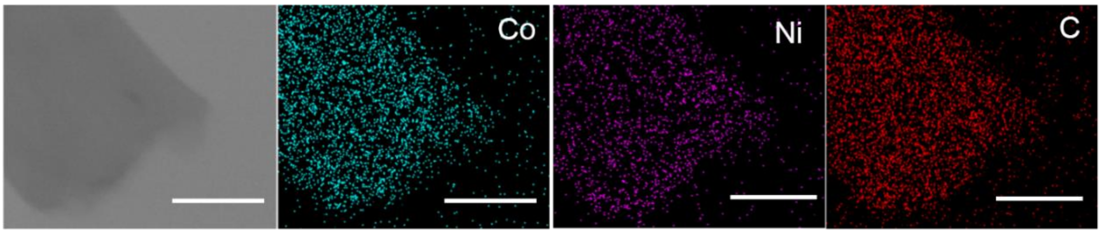

Supplementary Figure 47 | TEM image and element mapping of (Co, Ni)(BDC) nanosheets. Scale bar: 100 nm.

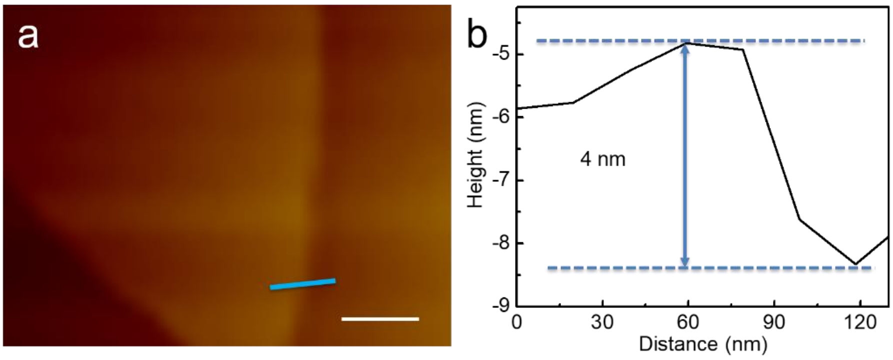

Supplementary Figure 48 | AFM image (a) and corresponding height profile (b) of (Co, Ni)(BDC) nanosheets. Scale bar: 100 nm.

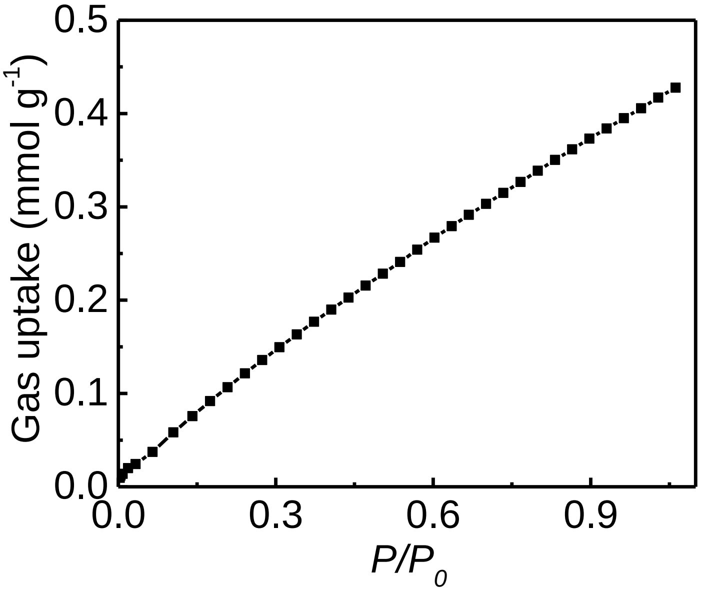

Supplementary Figure 49 | CO_2 adsorption isotherm for Co(BDC).

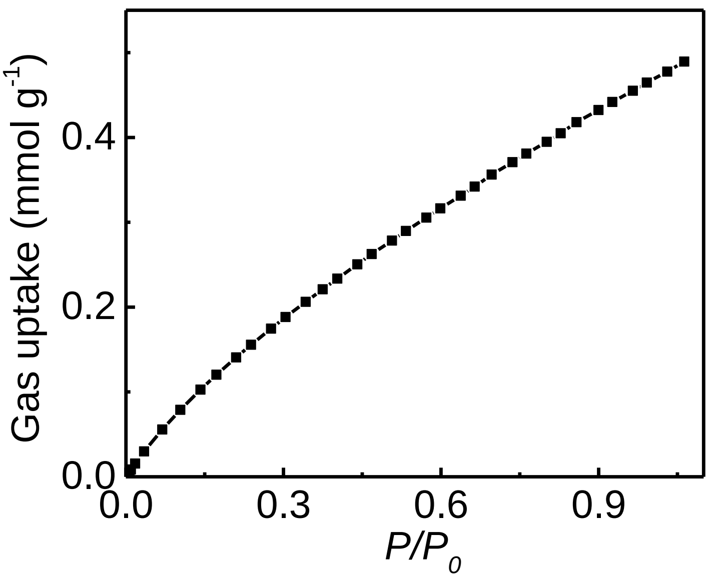

Supplementary Figure 50 | CO_2 adsorption isotherm for Cu(1,4-NDC).

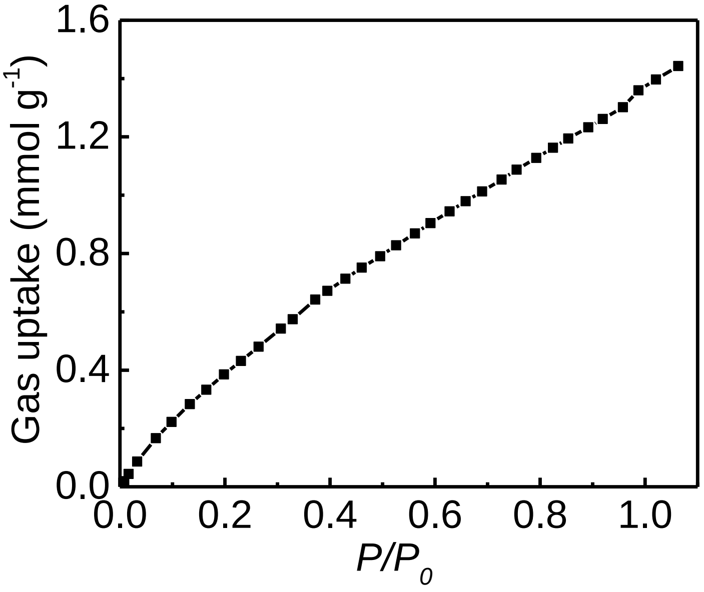

Supplementary Figure 51 | CO_2 adsorption isotherm for (Co, Ni)(BDC).

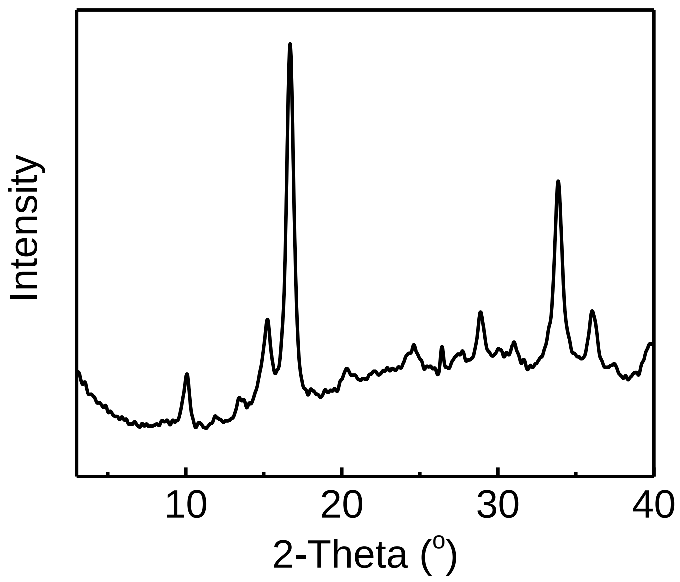

Supplementary Figure 52 | XRD pattern of the large-scale synthetic N-Cu(BDC). For the synthesis, $Cu(NO_3)_2 \cdot 3H_2O$ (1.8000 g), H_2BDC (1.7000 g), TEA (10 mL) and methanol (50 mL) were added into autoclave. Then CO_2 was charged into the autoclave under stirring to 7.38 MPa for 24 h at 35 °C. The precipitate was collected, washed by DMF and ethanol twice and dried at 80 °C in vacuum for 8 h.

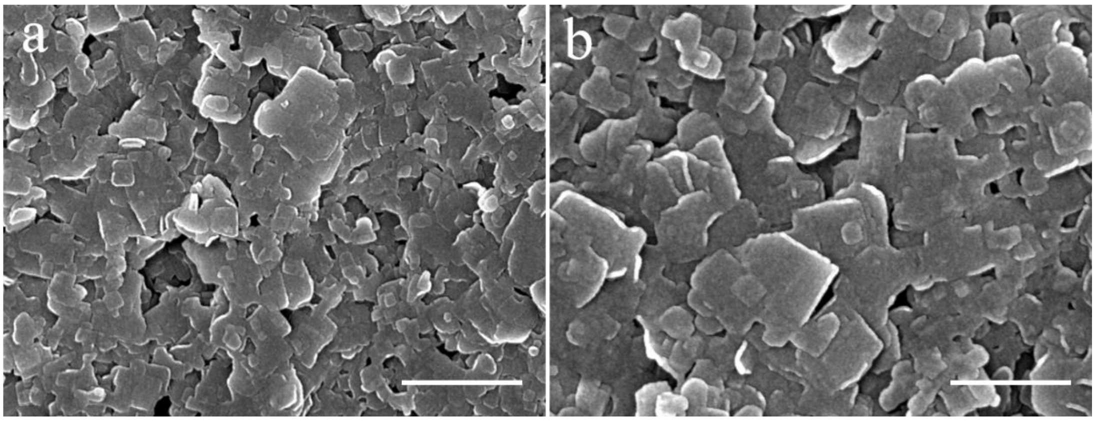

Supplementary Figure 53 | SEM images of the large-scale synthetic N-Cu(BDC).

Scale bars: 1 μm in a, 500 nm in b.

Supplementary Table 1 | EXAFS fitting parameters at the Cu K-edge for various samples.

Sample	Shell	N^{a}	R (Å) ^{b}	σ^2 (Å$^{-2}\cdot10^{-3}$) ^{c}	ΔE_0 (eV) ^{d}	R factor (%)
B-Cu(BDC)	Cu-O	5.0	1.96	5.9	7.0	0.8
	Cu-Cu	1.2	2.56	8.7	-9.0	
N-Cu(BDC)	Cu-O	4.0	1.96	4.7	6.8	0.7
	Cu-Cu	1.0	2.57	8.3	-9.0	

CN is the coordination number; *R* is interatomic distance (the bond length between central atoms and surrounding coordination atoms); σ^2 is Debye-Waller factor (a measure of thermal and static disorder in absorber-scatterer distances); ΔE_0 is edge-energy shift (the difference between the zero kinetic energy value of the sample and that of the theoretical model). *R* factor is used to value the goodness of the fitting. Error bounds that characterize the structural parameters obtained by EXAFS spectroscopy were estimated as N ± 20%; R ± 1%; σ^2 ± 20%; ΔE_0 ± 20%.

Supplementary Table 2 | Aerobic oxidation of benzyl alcohol to benzaldehyde catalysed by different MOFs.

Catalyst	Amount of MOF (mg)	Amount of benzyl alcohol (mg)	Amount of TEMPO (mg)	T (°C)	Time (h)	Yield (%)	TOF (mmol g^{-1} h^{-1})	Ref.
Cu_2BTEC nanowires [a]	30	20	28	75	4.5	98.1	1.27	4
Mesoporous $Cu_3(BTC)_2$ [b]	30	20	14	75	3	>90	1.91	5
Block $Cu_3(BTC)_2$	150	100	144	75	22	70	0.19	6
$Cu_3(BTC)_2$ [c]	30	20	28	75	7	100	0.82	7
Cu(BDC)-3.40 MPa	30	20	14	75	2.5	62	1.43	This work
Cu(BDC)-6.40 MPa	30	20	14	75	2.5	85	1.96	This work
Cu(BDC)-7.38 MPa	30	20	14	75	2.5	>99	2.30	This work
Cu(BDC)-8.60 MPa	30	20	14	75	2.5	>99	2.30	This work
Cu(BDC)-10.5 MPa	30	20	14	75	2.5	>99	2.30	This work

[a] $BTEC^{2-}$ 1,2,4,5-benzenetetracarboxylate; [b] BTC^{3-} 1,3,5-Benzenetricarboxylate; [c] $Cu_3(BTC)_2$ with core-shell microspheres.

Supplementary Table 3 | Aerobic oxidation of different alcohol to corresponding product catalysed by the N-Cu(BDC) synthesized in CO_2/methanol at 7.38 MPa and 35 °C with 0.1 mL of TEA for 24 h.

Entry	Substrate	Product	Time (h)	Yield (%)	TOF (mmol g^{-1} h^{-1})
1	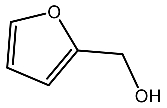	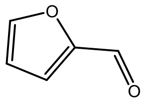	2.5	70	2.86
2	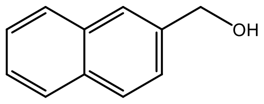	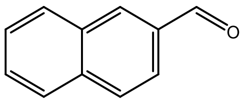	2.5	89	2.49
3	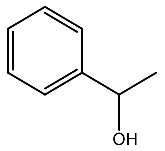	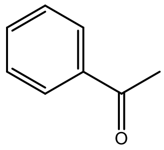	2.5	44	1.01
4	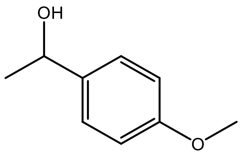	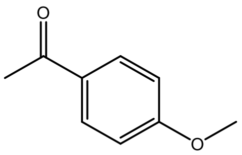	2.5	40	0.92

Reaction conditions: substrate 0.185 mmol, catalyst 30 mg, DMF 1 mL, TEMPO (0.5 equiv), Na_2CO_3 (1 equiv), 75 °C, oxygen atmosphere.

Supplementary References

1. Zhang, J. L. et al. Nanoemulsions induced by compressed gases. *Angew. Chem. Int. Ed.* **47**, 3012-3015 (2008).
2. Zhan, Y. J. et al. Synthesis of uniform hollow silica spheres with ordered mesoporous shells in a CO_2 induced nanoemulsion. *Chem. Commun.* **17**, 2365-2367 (2009).
3. Zhang, J. L. et al. Effect of compressed CO_2 on the size and stability of reverse micelles: Small-angle X-ray scattering and phase behavior study. *J. Chem. Phys.* **118**, 3329-3333 (2003).
4. Shi, J. B. et al. Rapid, room-temperature and template-free synthesis of metal-organic framework nanowires in alcohol. *ChemCatChem* **11**, 2058-2062 (2019).
5. Peng, L. et al. Highly mesoporous metal-organic framework assembled in a switchable solvent. *Nat. Commun.* **5**, 5465 (2014).
6. Dhakshinamoorthy, A. et al. Aerobic oxidation of benzylic alcohols catalyzed by metal-organic frameworks assisted by TEMPO. *ACS Catal.* **1**, 48-53 (2011).
7. Ju, S. et al. Simple transformation of hierarchical hollow structures by reduction of metal-organic frameworks and their catalytic activity in the oxidation of benzyl alcohol. *Chem. -Eur. J.* **25**, 7852-7859 (2019).
